# Heterogeneity and clinical significance of *ESR1* mutations in ER-positive metastatic breast cancer patients receiving fulvestrant

**DOI:** 10.1038/ncomms11579

**Published:** 2016-05-13

**Authors:** Jill M. Spoerke, Steven Gendreau, Kimberly Walter, Jiaheng Qiu, Timothy R. Wilson, Heidi Savage, Junko Aimi, Mika K. Derynck, Meng Chen, Iris T. Chan, Lukas C. Amler, Garret M. Hampton, Stephen Johnston, Ian Krop, Peter Schmid, Mark R. Lackner

**Affiliations:** 1 Genentech, Inc, South San Francisco, California 94080, USA; 2 Royal Marsden NHS Foundation Trust, London SW3 6JJ, UK; 3 Dana-Farber Cancer Institute, Boston, Massachusetts 02215, USA; 4 Barts Cancer Institute, Queen Mary University London, London EC1M 6BQ, UK

## Abstract

Mutations in *ESR1* have been associated with resistance to aromatase inhibitor (AI) therapy in patients with ER+ metastatic breast cancer. Little is known of the impact of these mutations in patients receiving selective oestrogen receptor degrader (SERD) therapy. In this study, hotspot mutations in *ESR1* and *PIK3CA* from ctDNA were assayed in clinical trial samples from ER+ metastatic breast cancer patients randomized either to the SERD fulvestrant or fulvestrant plus a pan-PI3K inhibitor. *ESR1* mutations are present in 37% of baseline samples and are enriched in patients with luminal A and *PIK3CA*-mutated tumours. *ESR1* mutations are often polyclonal and longitudinal analysis shows distinct clones exhibiting divergent behaviour over time. *ESR1* mutation allele frequency does not show a consistent pattern of increases during fulvestrant treatment, and progression-free survival is not different in patients with *ESR1* mutations compared with wild-type patients. *ESR1* mutations are not associated with clinical resistance to fulvestrant in this study.

## Abstract (web summary)

Fulvestrant degrades the oestrogen receptor. Here, the authors report on a clinical trial using fulvestrant and show that mutations in the oestrogen receptor alpha gene are prevalent in circulating tumour DNA and do not influence the clinical outcome of patients to fulvestrant.

## Graphical abstract

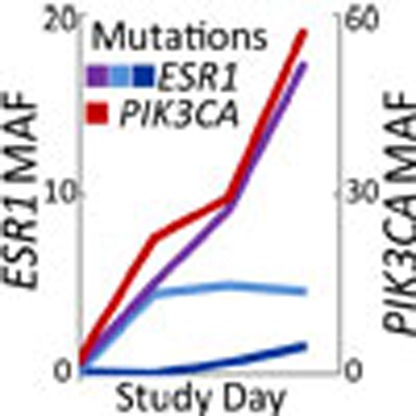

Oestrogen receptor alpha (ERα), encoded by the *ESR1* gene, is a member of the nuclear hormone receptor superfamily that is expressed in ∼70% of newly diagnosed breast cancers^1^. ERα and its cognate ligand oestrogen are the major drivers of tumour development and disease progression in luminal breast cancers, and agents that impair ER signalling in ER-positive breast cancers represent highly successful targeted therapies that are widely used in both early breast cancer, as well as the metastatic setting^2^. The selective oestrogen receptor modulator tamoxifen was approved in 1997 and still represents the standard of care for adjuvant treatment of early breast cancer^3^ especially in premenopausal patients, though aromatase inhibitors (AIs) that block biosynthesis of oestrogen are commonly incorporated into adjuvant treatment algorithms^4^ and represent a first-line standard of care in advanced, metastatic breast cancer in postmenopausal patients^5^. While the SERD fulvestrant is similar to selective oestrogen receptor modulators in antagonizing ER transcriptional activity, fulvestrant also causes ER degradation and has proven to be just as effective as AIs in advanced breast cancer^2^,^6^.

Despite the effectiveness of the various anti-hormonal therapies, intrinsic and acquired resistance remains a persistent problem that limits the ultimate effectiveness of these treatments^7^. The issue is particularly acute in the ER+ metastatic breast cancer (MBC) setting, where up to half of all patients show intrinsic resistance and do not benefit from therapy, and ultimately all ER+ MBC patients develop acquired resistance and progress on anti-hormonal therapies^5^. Preclinical and clinical investigations have demonstrated that these resistant ER+ breast cancers often retain ERα expression and dependence on oestrogen receptor signalling. In clinical practice, continued ER dependence is observed with patients who have progressed on one form of endocrine therapy^8^ often remaining responsive to different endocrine therapies in subsequent lines of therapy^9^. The discovery of acquired mutations in *ESR1* that confer ligand-independent and constitutive activation of ERα revealed one potential mechanism of resistance to endocrine therapies. *ESR1* mutations were originally reported in a small cohort of metastatic breast cancers in 1997 (ref. 10), and confirmed recently in several larger studies that utilized next-generation sequencing and suggested that such mutations are present in ∼20% of metastatic tissue samples but are generally not found in primary tumour samples^11^,^12^,^13^. The most frequently occurring *ESR1* mutations are in the ligand-binding domain (LBD) of ERα, generally clustering between amino acids 534–538, though mutations at other positions including S463 and E380 have also been described. Biochemical studies demonstrated that the Y537S mutation is constitutively active and results in full transcriptional activity in the absence or presence of oestrogen^14^, and subsequent reports demonstrated that other ERα LBD mutations similarly confer constitutive, ligand-independent activation^12^,^13^,^15^. Functional *in vitro* studies, along with the observation that mutations were found in metastatic tissue collected from patients who had undergone therapy with AIs, suggest a model wherein these mutations have an important role in acquired resistance to oestrogen deprivation mediated by AIs^16^.

An important unresolved question is whether ERα LBD mutations retain sensitivity to other endocrine agents, particularly SERDs such as fulvestrant that cause ERα degradation. *In vitro* findings have suggested that mutant ERα can still bind and be inhibited by fulvestrant, but that higher doses are required to inhibit mutant ERα compared with wild-type (WT) ERα^12^,^13^. Little is known as to how these mutations affect clinical outcomes in patients receiving endocrine therapies, and whether SERD treatment can achieve high enough clinical exposures to effectively degrade mutant ERα. The FERGI study (GDC4950g, NCT01437566) is a randomized phase 2 study that compared the pan-PI3K inhibitor pictilisib plus fulvestrant with placebo plus fulvestrant in patients with ER+ MBC who had received prior AI therapy^17^. As such, the FERGI study represents an excellent setting to analyse the prevalence of *ESR1* mutations in MBC patients that failed prior AI, as well as to explore the impact of these mutations on the therapeutic benefit of fulvestrant in these patients. In this study, baseline and on-treatment *ESR1* and *PIK3CA* mutation status was assessed in plasma samples from patients enrolled in the FERGI study. Several recent studies have shown the feasibility of detecting *ESR1* mutations in circulating tumour DNA (ctDNA) and that the mutation status in plasma DNA can more accurately reflect the extent of disease heterogeneity than the analysis of tissue from a single metastatic site^18^,^19^,^20^. In addition, ctDNA analyses offer the possibility of non-invasive monitoring of tumour mutation status over time and treatment, an approach that has been used successfully in the identification of circulating *KRAS* mutations in colorectal patients receiving anti-epidermal growth factor receptor (EGFR)-targeted therapies^21^, but which has not been well explored in the context of *ESR1* mutations and endocrine therapies. The major findings reported herein are that *ESR1* mutations are present in ∼40% of patients that have progressed on AI therapy and are numerically enriched in luminal A and *PIK3CA*-mutated tumours. Of the patients with a detectable *ESR1* mutation, 40% harbour multiple *ESR1* mutations, suggesting the convergent evolution of multiple AI-resistant clones. Serial monitoring of *PIK3CA* and *ESR1* mutations in plasma from 71 patients suggests that RECIST responses are associated with decreases in allele frequency (AF) for both *PIK3CA* and *ESR1*, but decreases in on-treatment AF did not reliably predict response since patients with stable or progressive disease (SD/PD) in some cases show decreases. In contrast, increases in on-treatment AF are only seen in patients with best response of SD or PD. Most importantly, *ESR1* mutations are not associated with differential progression-free survival (PFS) benefit in either the fulvestrant control arm or the experimental arm, suggesting that they may not be strongly associated with clinical resistance to SERD treatment.

## Results

### *ESR1* and *PIK3CA* mutations in ctDNA baseline plasma samples

**Figure 1:**
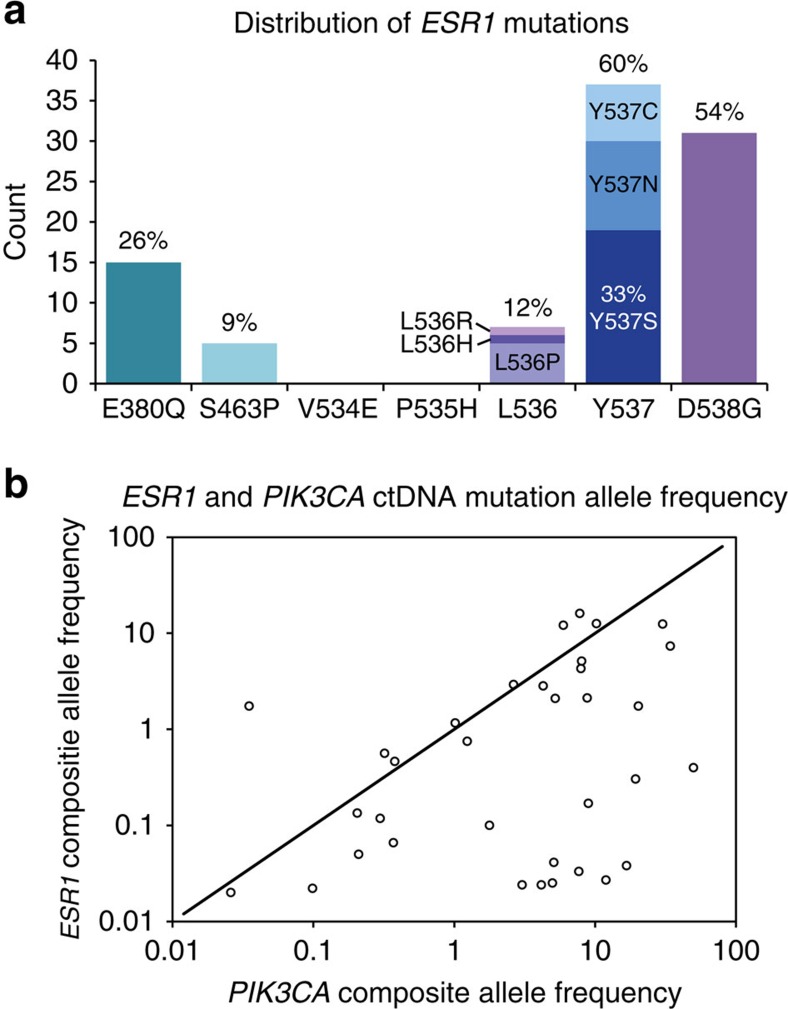
Distribution of *ESR1* mutations and AF. (**a**) Distribution of *ESR1* mutations detected in ctDNA from 153 part 1 patients. The bars indicate the number of patients for which each mutation was detected, and the percentage is relative to the total number of patients with mutations detected (57). Since multiple mutations were detected in 40% of the patients, the sum of the percentages is >100%. P535H and L536Q were not detected in any of the part 1 samples, but each was detected once among the 54 samples from part 2. V534E was not detected in any part 1 or part 2 sample. (**b**) Scatter plot of *ESR1* and *PIK3CA* ctDNA mutation allele frequency (AF) for patients where both mutations were detected (*n*=34). The mutation AF sum is plotted for patients with multiple *ESR1* or *PIK3CA* mutations.

**Figure 2:**
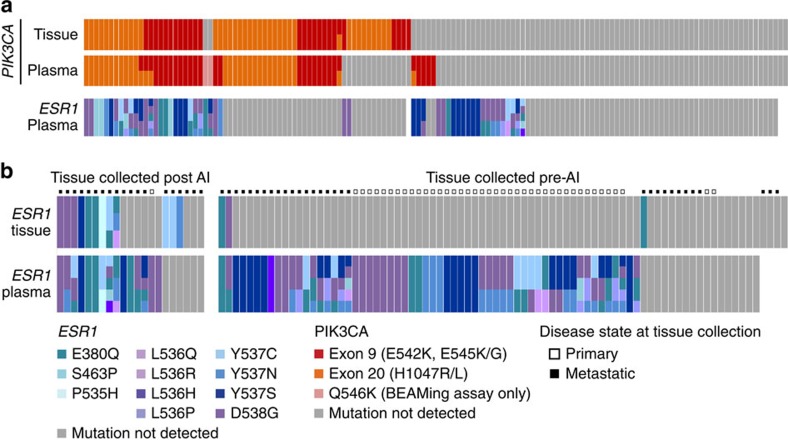
*PIK3CA* and *ESR1* mutation analysis of matched tissue and plasma samples. (**a**) *PIK3CA* and *ESR1* mutation analysis of matched tumour tissue and plasma samples from 142 patients from part 1 of the clinical study. Mutations are coloured according to exon (*PIK3CA*) or amino acid (*ESR1*). Grey indicates mutations assessed were negative, and blank indicates data not available. (**b**) *ESR1* tissue and matched plasma mutation status from 102 part 1 and part 2 patients. Patients whose tissue was collected after receiving AI therapy shown on the left (21), and those whose tissue was collected before AI on the right (81). Tissue collection at the time of primary or metastatic disease indicated with open or solid squares, respectively.

We used a sensitive and quantitative digital PCR assay to screen for 9 hotspot mutations in *PIK3CA* and 12 mutations in *ESR1* in ctDNA (see Methods). Table 1 shows *PIK3CA* and *ESR1* mutations as they relate to baseline clinical and demographic features, while Supplementary Table 1 shows the prevalence of *PIK3CA* and *ESR1* in breast cancer subsets. *PIK3CA* mutations were detected in ctDNA from 39.7% (62/156) of patients from the unselected phase of the study (part 1), which enrolled patients irrespective of *PIK3CA* tissue status (Table 1). The majority of patients (90.3%, 56/62) had only a single detectable *PIK3CA* mutation in ctDNA (Table 1). *ESR1* mutations were detected in ctDNA from 37.3% (57/153) of patients at baseline and exhibited markedly more heterogeneity than *PIK3CA* mutations. Among patients with any detectable *ESR1* mutation, 22.8% (13/57) had two mutations and 17.5% (10/57) had greater than two mutations. Mutations resulting in the amino-acid substitutions D538G, Y537S and E380Q were the most common alterations, found in 54%, 33% and 26% of *ESR1* mutant samples, respectively (Fig. 1a). In addition, the *PIK3CA* mutations had a higher median AF than *ESR1* (3.6% versus 0.45%) and samples with mutations in both genes generally showed a higher AF for *PIK3CA* compared with *ESR1* (Fig. 1b and Supplementary Fig. 1). Of the 11 *ESR1* mutations we detected in ctDNA, transiently expressed ERα proteins encoded by the most commonly detected mutations generally showed the strongest ligand-independent transcriptional activity *in vitro*, with the exception of E380Q that did not show significantly increased ligand-independent activity relative to WT ERα (Supplementary Fig. 2). Overall *PIK3CA* mutations in ctDNA were 86% concordant with tissue *PIK3CA* status determined by quantitative PCR assay (77% sensitivity and 93% specificity; Fig. 2a, Supplementary Table 2 and Supplementary Data 1). We also compared *ESR1* mutation status between tissue and plasma for a subset of patients and examined the relationship between the type of tissue (primary tumour or metastatic lesion) and collection time relative to AI therapy in the metastatic setting (Fig. 2b). We did not find any *ESR1* mutations in primary tumour tissue collected at diagnosis, consistent with previous reports. Moreover, we rarely found *ESR1* mutations in tissue that was collected before AI therapy (3/81 patients), but did detect *ESR1* mutations in tissue that was collected after progression on prior AI therapy in a much higher proportion of patients (12/21 patients). The most abundant *ESR1* mutation detected in plasma (that is, highest AF) was almost always the mutation detected in tissue (Supplementary Table 3). The overall concordance of *ESR1* tissue and ctDNA status was 31% when primary and metastatic samples are pooled together. The concordance between *ESR1* status in ctDNA and metastatic tissue was 47%, while concordance between ctDNA and primary tissue was 5%. Concordance was 57% and 23% for patients whose tumour tissue was collected after or before the administration of an aromatase inhibitor, respectively (Supplementary Table 4).

### Association of mutations with clinical features

We examined the relationship between plasma *ESR1* mutation status and a range of baseline clinical and demographic features. Overall, *ESR1* mutations occurred more frequently in patients who also harboured *PIK3CA* mutations, as 44.4% of patients with *PIK3CA* mutations detected in tissue also had a plasma *ESR1* mutation compared with 30.3% of patients without a *PIK3CA* mutation (Supplementary Table 1). Plasma *ESR1* mutations were also found in 44.4% of patients enrolled in part 2 of the FERGI study, which selected patients based on *PIK3CA* mutation tissue status (Supplementary Table 1, Supplementary Fig. 3 and Supplementary Data 1). The presence of an *ESR1* mutation in the plasma also showed a trend towards enrichment in patients with luminal A status determined by PAM50 algorithm^22^, as 41.4% of patients with PAM50 defined luminal A tumours had *ESR1* mutations compared with 31.8% of patients with luminal B tumours (Table 1 and Supplementary Table 1). A smaller numerical increase was seen in patients with PgR+ (38.5%) compared with PgR− (33.3%) tumours. Increases in plasma *ESR1* mutation prevalence were also associated with several clinicopathologic parameters. Sixty-one per cent of patients with *ESR1* mutations had visceral disease, whereas only 46.9% of *ESR1* WT patients had visceral disease (Table 1). In addition, 71.9% of patients with *ESR1* mutations had secondary resistance to prior AI therapy compared with 56.3% of *ESR1* WT patients, and 75.4% of patients with *ESR1* mutations had two or more metastatic sites compared with 56.3% of *ESR1* WT patients. Other demographic and clinical parameters were generally balanced between patients with *ESR1* mutations and *ESR1* WT patients, including age, race, region, Eastern Cooperative Oncology Group (ECOG) score, baseline tumour burden and others (Supplementary Table 5). In similar analyses, *PIK3CA* mutations in ctDNA generally were not associated with particular demographic and clinical features (Supplementary Table 6), with the exception of luminal status (Table 1). Similar to *ESR1*, a slightly higher proportion of patients with luminal A tumours had *PIK3CA* mutations in ctDNA compared with patients with luminal B tumours (44%, 44/100 versus 38%, 17/45).

### Association of baseline mutations with clinical outcome

**Figure 3:**
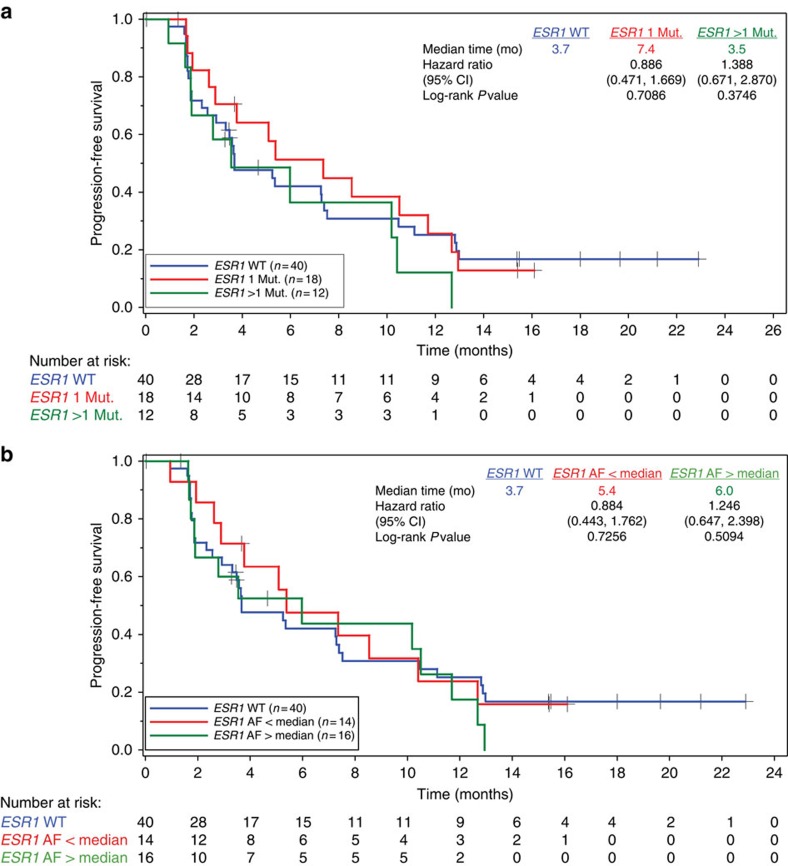
Kaplan–Meier plots of PFS in the fulvestrant and placebo part 1 patients. (**a**) PFS of *ESR1* mutation (Mut.) status divided by one (red) or greater than one (green) mutation, or WT (blue). (**b**) PFS by *ESR1* mutation AF less than (red) or greater than (green) the median AF. Hazard ratios are relative to WT for both plots. mo, months.

The FERGI study was a randomized phase 2 study comparing the pan-PI3K inhibitor pictilisib plus fulvestrant with placebo plus fulvestrant in patients with ER+ locally advanced or metastatic breast cancer, and so is an excellent setting to examine the association of *ESR1* and *PIK3CA* mutations in ctDNA with clinical outcome to these agents. *PIK3CA* status determined by quantitative PCR assay on archival tumour tissue was not associated with enhanced clinical benefit from the addition of pictilisib to fulvestrant in the FERGI study^17^. Here we retrospectively analysed whether *PIK3CA* and *ESR1* mutations detected in ctDNA were associated with differential benefit in either the control or the experimental arm of FERGI. Patients with a detectable plasma *ESR1* mutation did not show differential PFS in either the fulvestrant or fulvestrant plus pictilisib arm (Fig. 3 and Supplementary Fig. 4A). We also examined whether patients with more than one *ESR1* mutation (Fig. 3a) or a higher than median AF (Fig. 3b) showed differential outcome in the fulvestrant control arm, based on previous reports suggesting higher AFs of *BRAF* V600E mutations are associated with higher disease burden and shorter PFS to *BRAF*-targeted therapy^23^. Neither the presence of multiple *ESR1* mutations nor higher *ESR1* AFs were associated with a clear difference in risk of progression on fulvestrant (Fig. 3). Patients with a *PIK3CA* mutation in ctDNA had a PFS hazard ratio of 0.994 compared with *PIK3CA* WT patients in the fulvestrant control arm, and showed a PFS hazard ratio of 0.991 comparing *PIK3CA* WT patients in the fulvestrant plus pictilisib arm, with overlapping 95% confidence intervals (Supplementary Fig. 4B).

### Mutant AF changes and tumour response

**Figure 4:**
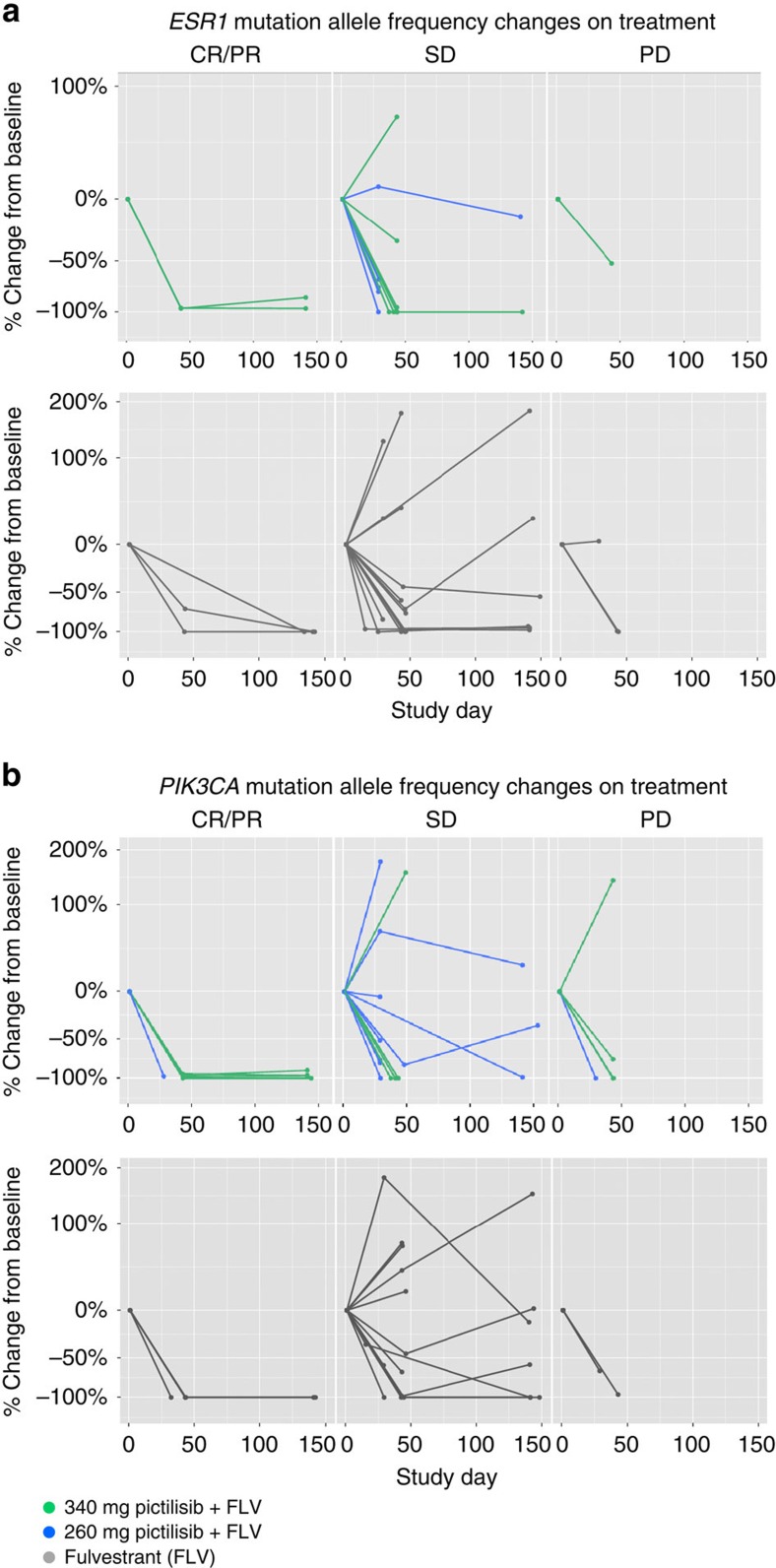
Serial analysis of ctDNA mutation AF changes binned by best clinical response. (**a**) *ESR1* and (**b**) *PIK3CA* ctDNA mutation AF changes while on part 1 (green) or part 2 (blue) pictilisib+fulvestrant or placebo+fulvestrant (grey) treatment arms. Patients are binned according to their best clinical response: CR/PR defined by RECIST criteria, and SD or PD as assessed by radiographic scans. Patients with a best response of non-CR/non-PD for patients without measurable disease at baseline are binned with SD. Each line represents one patient with the per cent change of AF relative to baseline (study day 0) plotted for two or three time points. The mutation AF sum is plotted for patients with multiple *ESR1* (**a**) or *PIK3CA* (**b**) mutations.

Longitudinal plasma samples from 71 patients were used to look at changes in AF over the course of treatment. Of these, 60 patients had at least one *ESR1* or *PIK3CA* mutation detected in at least one time point (Supplementary Fig. 5). Patients were binned based on their best clinical response irrespective of treatment, with complete or partial responses (CR/PR) defined by RECIST criteria, and stable disease (SD) or progressive disease (PD) as assessed by the investigator. Patients whose best response was CR or PR demonstrated robust decreases in *ESR1* and *PIK3CA* AF post treatment and did not show any evidence of increases in AF for either *ESR1* or *PIK3CA* at the time points monitored (Fig. 4a,b and Supplementary Fig. 6). In contrast, patients whose best response was SD or PD displayed more variable changes in AFs, with some mutations decreasing at cycle 2 but many others remaining unchanged or increasing at this time point. We also looked at whether *ESR1* mutation AF showed reproducible changes in the fulvestrant control arm, which might be predicted if these alterations resulted in structural changes in the protein that impaired the ability of fulvestrant to bind to ER and promote degradation of mutant ER protein. In fact, we found that a substantial majority of *ESR1* mutations in ctDNA showed decreases in AF at the initial time point and most of these decreases persisted at later time points (Fig. 4a and Supplementary Fig. 6). We also did not detect any trend towards particular *ESR1* hotspot mutations showing increases at any given time point (Supplementary Fig. 5), and did not detect the appearance of new *ESR1* mutations at later time points in the majority of patients. Specifically, of the 14 patients randomized to the fulvestrant control arm and on study for at least 140 days with no detectable *ESR1* mutations at baseline, 10 continued to be free of detectable plasma *ESR1* mutations. Two patients (1507 and 5801) showed the appearance of low-frequency *ESR1* mutations at cycle 6, one at D538G (0.12%) and the second with a D538G (0.09%) and Y537N (0.12%) mutation. Another two patients (1655 and 3501) had progressive disease and crossed over to the pictilisib plus fulvestrant arm and had detectable *ESR1* mutations at cycle 2 of this regimen, one with E380Q (0.13%) and the second with Y537C (0.08%).

### Intrapatient monitoring suggests clonal heterogeneity

**Figure 5:**
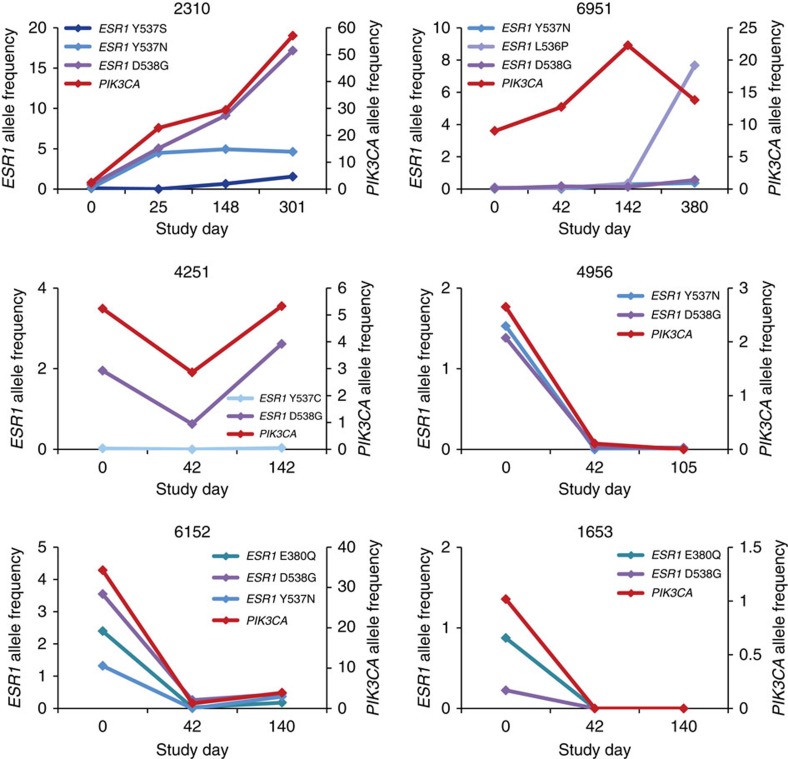
Clonal heterogeneity of *ESR1* mutations. Dynamic changes in ctDNA mutation AF changes for six patients while on treatment as examples of clonal heterogeneity of *ESR1* mutations. Each line represents a different *ESR1* or *PIK3CA* mutation, and raw AF values are plotted for each time point. Patients 2310 and 6951 demonstrate divergent behaviour of mutations. Patients 4956, 4251, 6152 and 1653 demonstrate consistent increases and/or decreases of AF.

Intrapatient patterns of AF changes over time were examined in all 60 patients with longitudinal data who had at least one detectable mutation. The complete data set is shown in Supplementary Fig. 5, and we highlight several patients that show utility for monitoring differential response to therapy based on clonal heterogeneity (Fig. 5). Patient 2310 showed evidence of a *PIK3CA* mutation that started at relatively low AF markedly increased over treatment. A concomitant D538G mutation in *ESR1* tracked well with the *PIK3CA* mutation over time and treatment. However, additional *ESR1* mutations Y737S and Y737N showed divergent behaviour and did not increase over time. Patient 6951 showed evidence of a *PIK3CA* mutation that increased in AF over time, while concomitant *ESR1* mutations either remained unchanged or showed a slight increase, but with a different time course from the *PIK3CA* mutation. Other patients had multiple mutations and showed consistent changes over time, such as patients 4956, 1653 and 6152, who had multiple mutations and showed consistent decreases for all observed mutations.

## Discussion

Recent studies have shown that *ESR1* mutations can be detected in metastatic ER+ breast cancer tissues but generally not in primary tumour tissues collected before the initiation of endocrine therapy. Initial prevalence estimates varied widely, ranging from 12% (9/76) in an overall population of metastatic ER+ breast cancers^11^ to 55% (6/11) in a small cohort of patients selected based on having had multiple rounds of hormonal therapy^12^. These tissue-based findings have been extended to ctDNA in several small studies of limited numbers of patients, and recently in a cohort of 171 patients receiving AI therapy^24^. Guttery *et al*.^19^ analysed ctDNA from 48 ER+ breast cancer patients receiving a variety of therapies and showed that they could detect *ESR1* mutations in 11 patients, while Sefrioui *et al*.^20^ analysed ctDNA and matched tissue from 7 ER+ patients and detected mutations in ctDNA in 4 of 6 patients with tumour mutations. Recently, Wang *et al*.^25^ showed that they could detect *ESR1* mutations in 25% of advanced breast cancer patients (9/29), and that changes in AF could be monitored longitudinally. Schiavon *et al*.^24^ recently showed convincingly that *ESR1* mutations in ctDNA are rarely acquired during adjuvant AI treatment but are commonly selected by AI therapy for metastatic disease. To our knowledge, this report is the first to examine *ESR1* mutations in a large, clinically well-defined cohort of patients who all received prior AI therapy, followed by the SERD fulvestrant in the context of a randomized trial.

These findings regarding the prevalence and overlap of *PIK3CA* and *ESR1* help define an emerging model of polyclonal breast tumour evolution under the selective pressure of endocrine therapy. Genome-wide ‘phylogenetic' analysis of breast cancer through next-generation sequencing has suggested that most *PIK3CA* mutations occur early in the process of tumour development and generally are associated with a tumour's dominant subclonal lineage^26^. In contrast, *ESR1* mutations have been proposed to arise late and in a subclonal manner, since these mutations are generally not detected in primary tumour samples but only in metastatic lesions^11^,^13^,^16^. We found that *PIK3CA* mutations in ctDNA occurred on average at 10-fold higher allelic frequencies than *ESR1* mutations and generally each patient had a single mutation in *PIK3CA*. In contrast, *ESR1* mutations were often polyclonal, as 40% of patients had more than one *ESR1* mutation and in rare cases patients had four or five detectable low-frequency *ESR1* mutations. Most patients in the study submitted only primary archival tissue for analysis, but a subset had metastatic biopsies available. Analysis of matched metastatic tumour tissue and ctDNA showed that while generally a single mutation could be detected in metastatic biopsies, matched ctDNA often showed additional mutations beyond the one identified in the tumour sample. These results suggest that mutational analysis of ctDNA may offer a superior platform to survey intrapatient mutational heterogeneity, likely by virtue of integrating data from diverse metastatic lesions, whereas analysis of one site may not provide an accurate picture of the overall genomic landscape.

Our data also provide a comprehensive survey of the prevalence of *ESR1* mutations in advanced ER+ metastatic breast cancer patients who have received prior AI therapy, as prior AI treatment was an enrollment criterion for the FERGI study. Overall, *ESR1* mutations were detected in ctDNA from 37.3% of patients enrolled in part 1 of the clinical study. We found that *ESR1* mutations showed a trend towards higher prevalence in patients with tumours classified as luminal A by PAM50 compared with luminal B (41.4 versus 31.8%), as well as in patients with tumours harbouring *PIK3CA* mutations (44.4%). *ESR1* mutations were also found in 44.4% of patients enrolled in the *PIK3CA* selected part of the FERGI study. These findings are consistent with a model wherein more oestrogen-dependent, luminal A tumours are particularly susceptible to selective pressure to maintain ER signalling in the presence of oestrogen depletion, and that acquisition of *ESR1* mutations is a highly prevalent mechanism of resistance. Intriguingly, exploratory *post hoc* subgroup analysis in the FERGI study suggested improvement in PFS in patients with ER+ and PgR+ tumours treated with pictilisib plus fulvestrant (median of 7.2 months in the combination arm versus 3.7 months in the control arm)^17^. The finding that *ESR1* mutations are numerically enriched in luminal A, *PIK3CA*-mutated and PgR+ tumours, together with the FERGI clinical results, may indicate that highly endocrine sensitive tumours are particularly sensitive to combined targeting of ER and PI3K signalling, a hypothesis that should be explored in future clinical studies.

The careful annotation of tumour collection date relative to AI therapy also allowed us to ask whether mutations were specifically associated with prior AI therapy. Indeed, we found that mutations were very infrequently detected in metastatic samples collected before AI therapy, but were common in patients where tissue was collected after the initiation of AI therapy, supporting the notion that these particular *ESR1* mutations arise in the setting of AI exposure as a means of conferring ligand-independent oestrogen receptor signalling to tumour cells. These findings are consistent with recent work from Schiavon and colleagues, which suggested that *ESR1* mutations are found exclusively in oestrogen receptor-positive breast cancer patients previously exposed to AI, and that patients with *ESR1* mutations had a substantially shorter PFS on subsequent AI-based therapy^27^. Overall, the data suggest a model of convergent evolution, wherein multiple metastatic subclones acquire independent activating mutations in *ESR1* as a means of maintaining oestrogen signalling in the presence of anti-endocrine therapies. These data also have implications in terms of understanding the functional significance of the various hotspot mutations in *ESR1*. While the distribution of *ESR1* mutations at the hotspot residues Y537 and D538 was consistent with previous reports, we found a slightly higher than expected distribution (26% of patients with *ESR1* mutations detected in plasma) of the N-terminal LBD mutation E380Q. While previous studies report increased sensitivity of this mutant to low concentrations of oestrogen and varying degrees of oestrogen-independent activity of this mutant *in vitro* in ER-negative models^15^,^28^, its functional significance *in vivo* remains unknown. We do not find significant ligand-independent activity of this mutant in ER-negative cells *in vitro*, consistent with studies from an *ESR1* E380Q patient-derived xenograft model^15^; however, our finding that the E380Q mutation occurred in two breast cancer patients' tissues and in one case at a high AF (18.1%), and further, one of these samples were collected before the administration of AI therapy suggest a functional role of this mutation in ER+ breast cancer independent of AI resistance. Some clues to its mechanistic role come from a recent study showing that in contrast to the Y537S and D538G mutants, the *ESR1* E380Q mutant cannot bind the ERα co-regulatory protein prohibitin-2 (PHB2), which directly represses the transcriptional activity of activated ERα^29^. These data suggest a possible mechanism whereby the E380Q mutant could enable increased ER signalling or prolonged ER signalling in the presence of oestrogen by virtue of its inability to bind the co-repressor PHB2, and our findings are consistent with this hypothesis.

Recent studies have highlighted the ability of mutational analysis of ctDNA to serve as a potential surrogate for antitumour activity and for therapeutic monitoring^30^. Dawson *et al*.^31^ used a next generation sequencing (NGS)-based approach to derive personalized panels of assays that could be used to monitor response over time and treatment, and found that circulating tumour DNA levels showed a greater dynamic range, and greater correlation with changes in tumour burden, than did CA 15-3 or circulating tumour cells, in advanced breast cancer patients. Similarly, Frenel *et al*.^32^ used serial next-generation sequencing of circulating ctDNA to monitor mutational response to a collection of targeted agents in patients from phase I studies, and found that the monitoring of mutation AF in consecutive plasma samples during treatment demonstrated potential treatment-associated clonal responses. Our results are broadly consistent with these studies but highlight some of the challenges associated with using ctDNA mutation status as a surrogate marker of antitumour activity. We found that patients that had radiographically confirmed RECIST responses (CR or PR) uniformly showed substantial decreases in both plasma *PIK3CA* and *ESR1* AF, and that these changes were apparent at the earliest time point we assessed. However, we also observed plasma *PIK3CA* and *ESR1* AF decreases of similar magnitude in a substantial fraction of patients whose best response was stable disease or progressive disease. This finding suggests a speculative model in which certain lesions in these patients were potentially responding to these therapies based on the decreased AFs, and possibly that progression was due to growth of other lesions that did not harbour the specific mutations being tracked. Analysis with multiplexed panels, perhaps based on NGS platforms, may be necessary to interrogate and monitor overall tumour status and response to therapy. In contrast, increases in plasma *PIK3CA* or *ESR1* AFs were only seen in patients with stable disease or progressive disease, suggesting some potential utility in using the increase of mutation AFs to predict reduced clinical benefit if such findings can be confirmed in future prospective studies.

Our findings also have important implications for the treatment of patients with acquired *ESR1* mutations. Previous studies have not addressed the impact of treatment with the SERD fulvestrant on PFS in patients with baseline *ESR1* mutations. Our study does not provide any evidence that patients with plasma mutations in *ESR1* have differential PFS with fulvestrant treatment, compared with patients without *ESR1* mutations, suggesting *ESR1* mutations in aggregate may not be associated with innate or acquired resistance to fulvestrant. Previous *in vitro* studies showing reduced sensitivity of the constitutively active mutants Y537S and D538G to fulvestrant suggested potential clinical resistance of *ESR1* mutant patients to clinically achievable fulvestrant exposures^11^,^12^,^13^,^33^. These studies consistently showed that high suprapharmacologic concentrations of fulvestrant were required to inhibit transcriptional activity of ERα mutants. Of note, these experiments were primarily conducted in ER-negative cell line models, and it is possible that ERα mutants may behave differently on dimerization with WT ERα and in transcriptional complex with co-regulator proteins expressed in ER+ breast cancers. Indeed, when these same mutations were expressed in two ER+ cell lines, fulvestrant could downregulate WT and mutant ERα protein levels, and could partially suppress growth of the mutant-expressing cells^15^. Thus, the concentrations of fulvestrant needed to inhibit mutant ERα activity may differ in the ER+ versus ER− cell line setting, and effective clinical exposures of fulvestrant to address mutant ERα may have been achieved in the FERGI study of ER+ breast cancer patients, although the data presented were an exploratory *post hoc* analysis. Whether additional *ESR1* mutations beyond the 12 evaluated in our study could alter the structure of ERα and impair its degradation by fulvestrant, impacting therapeutic benefit from fulvestrant, remains to be determined. We also examined whether patients with more than one mutation, or a higher AF, potentially indicative of higher disease burden, showed longer PFS and again did not find any relationship between these parameters and clinical outcome. Moreover, serial analysis of mutations over time did not reveal the consistent appearance of *ESR1* mutations at later time points, and in fact, *ESR1* mutations in general showed a pattern of decreased AFs in most patients. This is in contrast to resistance mechanisms in other disease settings, such as *KRAS* mutations in patients treated with anti-EGFR antibodies, where a clear pattern of increases in *KRAS* in ctDNA occur post treatment and which are associated with disease progression^21^,^34^. Caution is required interpreting these findings since they are retrospective in nature and derived from exploratory subsets of a phase 2 trial, but our findings suggest that SERDs may have activity in patients with *ESR1* mutations who are refractory to AI therapy and that prospective testing of this hypothesis is warranted. Next-generation orally available SERDs designed to more completely downregulate and degrade ERα are currently in clinical development, and our findings can inform the design of future clinical trials involving fulvestrant as well as these next-generation SERDs^35^,^36^. It will be of great interest to determine if these inhibitors are efficacious in patients harbouring multiclonal *ESR1* mutations, ideally through prospective stratification with a validated diagnostic assay optimized for ctDNA.

## Methods

### Patient population

Study GDC4950g was an international, multicentre, randomized, double-blinded, placebo-controlled, phase 2 clinical trial evaluating the combination of pictilisib plus fulvestrant versus placebo plus fulvestrant in AI-resistant locally advanced or MBC^37^. The study was conducted in two parts. In part 1, eligible patients were post-menopausal women aged ≥18 years with ER+/HER2– locally advanced or MBC appropriate for fulvestrant treatment based on national or local treatment guidelines, relapsed during or within 6 months of AI treatment in the adjuvant setting, or with PD during treatment with an AI in the metastatic setting. The most recent treatment before enrollment was required to be an AI, with a minimum duration of 4 weeks of treatment before recurrence or PD. In part 1 of the study, 168 patients were randomized (1:1) to receive 28-day cycles of fulvestrant 500 mg on days 1 and 15 of cycle 1 and day 1 of subsequent cycles with either pictilisib 340 mg QD (*n*=89) or matching placebo (*n*=79). Part 1 also enrolled 32 patients to receive the PI3K/mTOR inhibitor GDC-0980, but this arm was discontinued based on safety data and the recommendation of the Internal Monitoring Committee. The primary analysis was based on a 6-month median duration follow-up. Inclusion criteria for part 2 were similar to those for part 1, except that in part 2 all patients were required to have a *PIK3CA*-mutated tumour and the requirement for an AI to be the last therapy was removed. Part 2 randomized (2:1) 61 patients to receive fulvestrant with either pictilisib 260 mg QD (*n*=41) or placebo (*n*=20). The study was conducted in accordance with Good Clinical Practice guidelines and the Declaration of Helsinki. Written informed consent was obtained from all patients before enrollment, in agreement with approved protocols from respective ethics committees at every site.

### Patient plasma and tissue samples

Baseline plasma samples for ctDNA analysis were collected from 156 of the 168 randomized part 1 patients. ctDNA was tested for *PIK3CA* mutations in all 156 samples, of which 142 had matched *PIK3CA* mutation status from tumour tissue for specificity and sensitivity analysis. ctDNA was tested for *ESR1* mutations from 153 of 156 patients, and an additional 24 patients from the GDC-0980 arm. ctDNA was also tested for *PIK3CA* and *ESR1* mutations from 60 and 54 part 2 patients, respectively. *PIK3CA* and *ESR1* ctDNA prevalence and associations with patient demographics and molecular characteristics were based on the 156 or 153 part 1 patients, respectively. Residual plasma samples from pharmacokinetic analysis were used for ctDNA analysis from 71 patients (49 from part 1 and 22 from part 2) that consented to the use of the residual material for exploratory biomarker analyses. These samples were collected at cycle 2 day 15 (part 1) or cycle 2 day 1 (part 2), cycle 6 day 1 and crossover cycle 2 day 15 (for patients that received crossover therapy). Tumour tissue was tested for *ESR1* mutations from a total of 102 patients, 64 and 27 from part 1 and part 2, respectively, and an additional 11 patients from the GDC-0980 arm. Patient tissue samples were chosen based on the presence of an *ESR1* mutation in the baseline plasma sample or if the tissue was collected after the patient was diagnosed with metastatic disease. Two tissue samples tested for *ESR1* mutations were from fresh biopsies collected within 3 weeks of the blood collection, and the remaining tissue samples were archival.

### Tissue mutation testing

Genomic DNA was isolated from formalin-fixed paraffin-embedded tumour tissue and tested for *PIK3CA* mutation status by quantitative real-time PCR for activating missense mutations C420R, E542K, E545A/G/K and H1047L/R/Y at a central laboratory. Droplet digital PCR probe assays were designed to assess *ESR1* mutation status for 10 mutations: E380Q (1508 G>C); P535H (1974 C>A); L536H (1977T>A); L536P (1977T>C); L536Q (1977-8 TC>AG); L536R (1977T>G); Y537C (1980A>G); Y537N (1979T>A); Y537S (1980A>C); and D538G (1983A>G). For patient samples with sufficient DNA available (*n*=60), all mutations detected in plasma (excluding *ESR1* S463P) were assessed in the matched tissue. For patients with limiting DNA available (*n*=15), only the one or two mutations with the highest allele fractions detected in plasma were assessed in tissue. For patient samples with no mutation detected in plasma, all mutations were assessed in matched tissue samples if there was sufficient DNA (*n*=17) or only the most common mutations (Y537C/N/S, D538G and E380Q) if there was limited DNA (*n*=10). An *ESR1* WT assay containing a 5′ HEX-labelled probe was used as a reference to estimate WT copy number and to calculate mutant AF in each individual sample. *ESR1* mutant assays containing a 5′ FAM-labelled probe were designed to use the identical forward and reverse primer sequences as the WT assays and to differ only in the probe sequences. *ESR1* assay sequences are listed in Supplementary Table 7. All assays were performed on the QX200 Droplet Digital PCR System (BioRad) according to the manufacturer's protocol and using an annealing temperature of 55 °C. Each assay run included *ESR1* WT and mutant oligonucleotide controls and a human WT genomic DNA control (Roche) to determine fluorescence amplitude thresholds for positive and negative mutation calls. To achieve a sensitivity level of ∼0.02% mutant AF per individual *ESR1* mutation, 20 ng genomic DNA was assessed per mutation. *ESR1* mutant AFs were calculated using QuantaSoft software (BioRad).

### Plasma mutation testing

ctDNA was tested for *PIK3CA* and *ESR1* mutations using the OncoBEAM BC1 BEAMing Digital PCR panel (conducted at Sysmex, Hamburg, Germany), as previously described^38^. Briefly, DNA (3–1,500 ng) was isolated from 1 to 2 ml of plasma and pre-amplified in a PCR reaction using gene specific primers with a tag sequence. The pre-amplified DNA was then subjected to an emulsion PCR containing tag specific primers bound to magnetic beads. The resulting beads with bound amplified DNA were hybridized to WT or mutant specific fluorescent probes followed by flow cytometry to quantitate the fraction of mutant alleles to total DNA alleles. The assay detects 9 *PIK3CA* mutations (C420R, E542K, E545K/G, Q546K, M1043I and H1047R/L/Y) and 12 *ESR1* mutations (E380Q, S463P, V524E, P535H, L536H/P/Q/R, Y537C/N/S and D538G), and has a limit of detection of 0.02% assuming the DNA input is at least 16 ng. Approximately 55% of all plasma samples tested had sufficient DNA for this limit of detection, and 97% of the samples had sufficient DNA to detect 0.06%. Unless otherwise noted, all reported mutant allele fractions are a composite of all *ESR1* or *PIK3CA* mutant allele fractions for a given sample, if the sample has two or more *ESR1* or *PIK3CA* mutations detected.

### Gene expression

Haematoxylin–eosin sections were prepared for all samples and were reviewed by a pathologist to confirm diagnosis and assess tumour content. RNA extraction and gene expression analysis was performed as previously described^39^. Briefly, FFPE sections were macrodissected to enrich for neoplastic tissue followed by RNA extraction using the High Pure FFPE RNA Micro Kit (Roche Applied Sciences). Gene expression was subsequently determined using the NanoString nCounter Analysis System on a custom gene panel which includes 50 genes from the PAM50 breast cancer intrinsic classification signature^22^, listed in Supplementary Table 8. A total of 200 ng of RNA was hybridized to the codeset overnight at 65 °C according to the NanoString protocol. Samples were loaded onto the NanoString nCounter Prep Station and read using the NanoString nCounter Digital Analyzer. Raw expression data was then log2-transformed and normalized against included housekeeping genes.

### Breast cancer molecular classification

PAM50 subtype prediction was carried out using a random-forest-based classifier that was derived from an independent training set of 157 breast cancer samples (data not shown) and 50 genes from the PAM50 signature^22^. We assigned PAM50 subtypes for the training samples based on consensus calls from both public nearest-centroid-based PAM50 classification and hierarchical clustering approaches to reduce platform and population biases. Among them, 112 samples had consistent calls from both approaches and formed the final training set. A random-forest-based classifier was then developed with an estimated out-of-bag error rate of 7.1% and applied to predict new samples.

### Data availability

The authors declare that the data supporting the findings of this study are available within the article and its Supplementary Information files.

## Additional information

**How to cite this article:** Spoerke, J. M. *et al*. Heterogeneity and clinical significance of *ESR1* mutations in ER-positive metastatic breast cancer patients receiving fulvestrant. *Nat. Commun.* 7:11579 doi: 10.1038/ncomms11579 (2016).

## Supplementary Material

Supplementary Information Supplementary Figures 1-6 and Supplementary Tables 1-8.

Supplementary Data 1 ESR1 plasma status and PIK3CA tissue plasma status for parts 1 and 2

## Figures and Tables

**Table 1:** Baseline ctDNA mutation prevalence and patient subsets.

Part 1: randomized GDC-0941 and placebo GDC-0941 patients			
	*ESR1* wild type, 96 (62.7%)	*ESR1* mutation, 57 (37.3%)	Total, 153 (100%)
*ESR1* mutation number			
0	96 (100%)	0	96 (62.7%)
1	0	34 (59.6%)	34 (22.2%)
2	0	13 (22.8%)	13 (8.5%)
3+	0	10 (17.5%)	10 (6.5%)
			
*PIK3CA* status ctDNA			
Wild type	68 (70.8%)	23 (40.4%)	91 (59.5%)
Mutation	28 (29.2%)	34 (59.6%)	62 (40.5%)
			
Luminal status			
Luminal A	58 (60.4%)	41 (71.9%)	99 (64.7%)
Luminal B	30 (31.3%)	14 (24.6%)	44 (28.8%)
Other	8 (8.3%)	2 (3.5%)	10 (6.5%)
			
Visceral disease			
Yes	45 (46.9%)	35 (61.4%)	80 (52.3%)
No	51 (53.1%)	22 (38.6%)	73 (47.7%)
			
Resistance to prior AI			
Primary	42 (43.8%)	15 (26.3%)	57 (37.3%)
Secondary	54 (56.3%)	41 (71.9%)	95 (62.1%)
Unknown	0	1 (1.8%)	1 (0.7%)
			
Number of metastatic sites			
0	5 (5.2%)	1 (1.8%)	6 (3.9%)
1	37 (38.5%)	13 (22.8%)	50 (32.7%)
2+	54 (56.3%)	43 (75.4%)	97 (63.4%)
			
	***PIK3CA* wild type, 94 (60.3%)**	***PIK3CA* mutation, 62 (39.7%)**	**Total, 156 (100%)**
*PIK3CA* mutation number			
0	94 (100%)	0	94 (60.3%)
1	0	56 (90.3%)	56 (35.9%)
2+	0	6 (9.7%)	6 (3.8%)
			
Luminal status			
Luminal A	56 (59.6%)	44 (71.0%)	100 (64.1%)
Luminal B	28 (29.8%)	17 (27.4%)	45 (28.8%)
Other	10 (10.6%)	1 (1.6%)	11 (7.1%)
