# Supplementary material for: Heterogeneity and clinical significance of ESR1 mutations in ER-positive metastatic breast cancer patients receiving fulvestrant
Source: Nat Commun. 2016 May 13;7:11579. doi: 10.1038/ncomms11579 (PMC4869259; doi:10.1038/ncomms11579)
Supplement: Supplementary Information — Supplementary Figures 1-6 and Supplementary Tables 1-8. [file ncomms11579-s1.pdf]

Supplementary Figure 1
Distribution of mutant allele frequencies

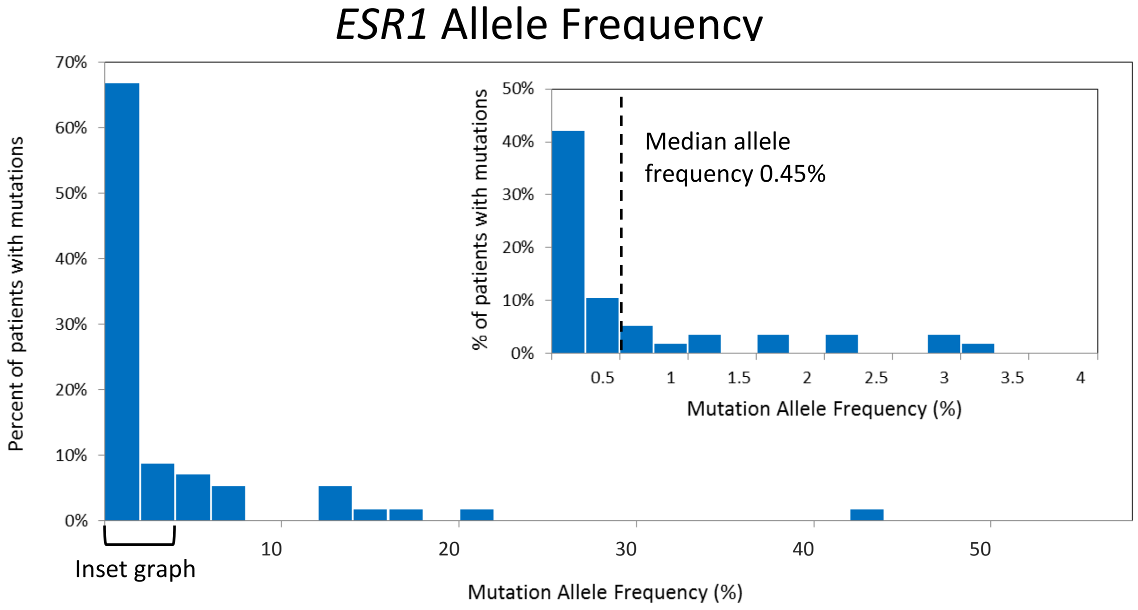

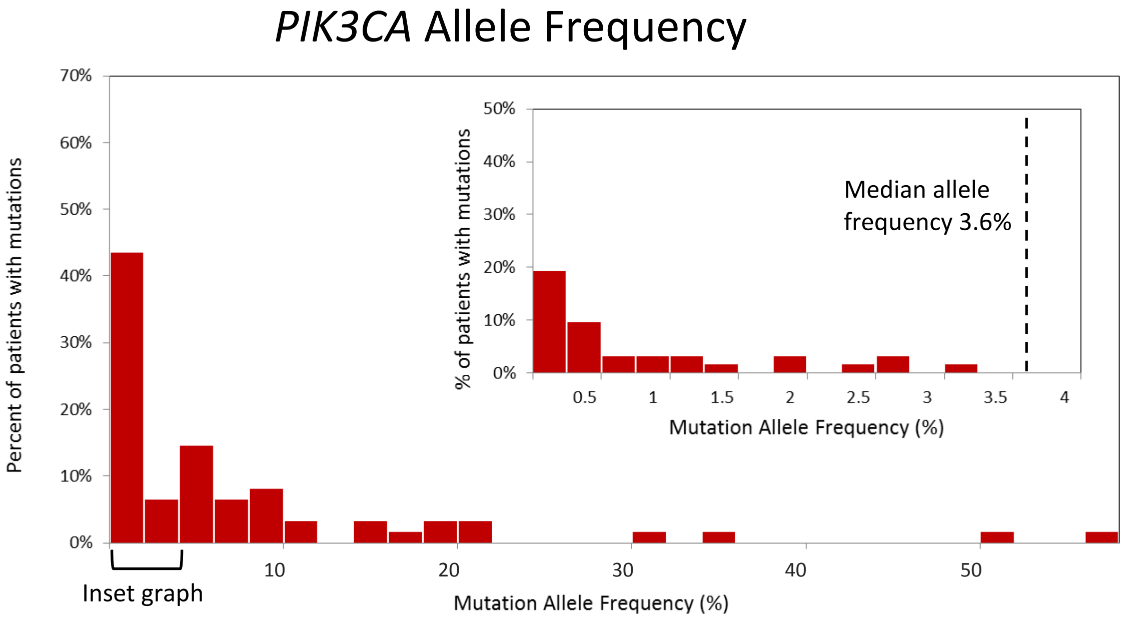

Supplementary Figure 1 Histogram of *ESR1* (blue) and *PIK3CA* (red) ctDNA mutant allele frequencies for part 1 patients (ESR1 n=57, PK3CA n=62). Each bar represents the percent of patients with a mutation allele frequency within the specified bin, for example 0.02% to 2% for the first bin, >2% to 4% for the second bin, etc. The inset graphs are histograms for patients with mutant allele frequencies less than 4%, which represents half or more of the patients for both *ESR1* and *PIK3CA*.

Supplementary Figure 2

Estrogen Response Element (ERE) reporter assay indicates relative levels of ligand-independent transcriptional activity of ERα mutants

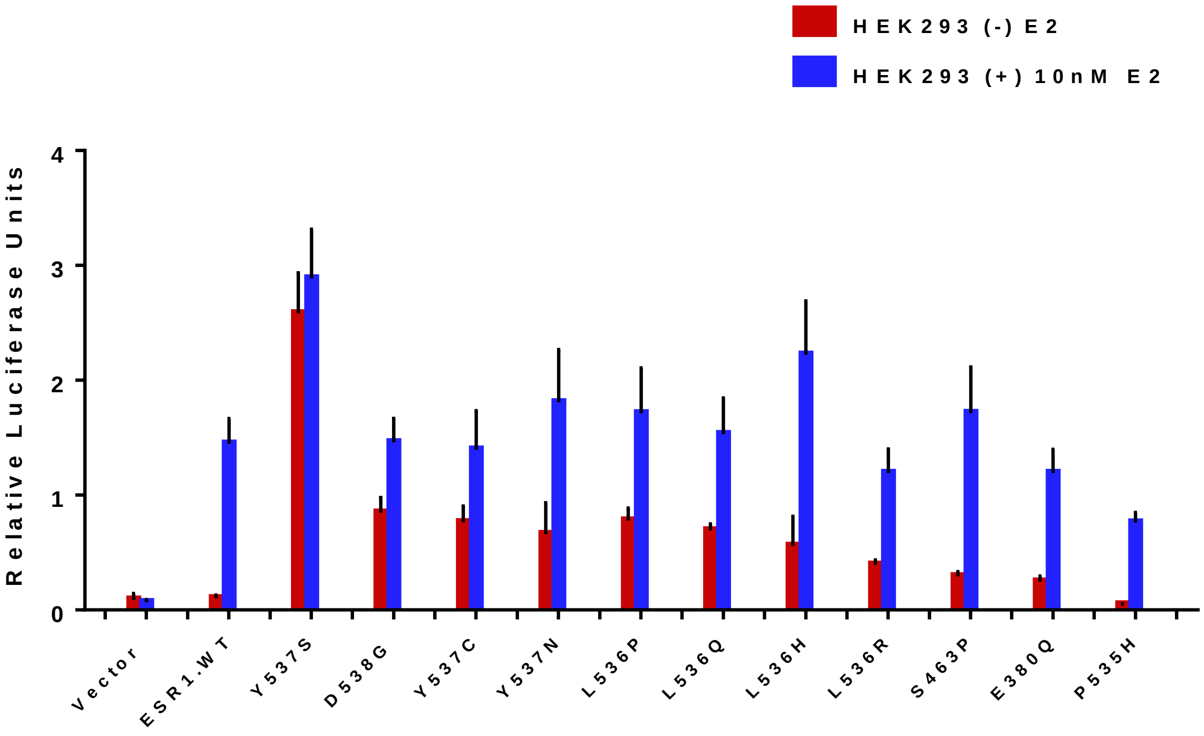

Supplementary Figure 2

Relative ERE transcriptional activity levels of ERα proteins. HEK293 cells obtained from ATCC were cotransfected with an ERE–firefly luciferase reporter plasmid, a plasmid constitutively expressing Renilla luciferase as an internal control, and pcDNA3.1+ constructs containing WT ESR1 cDNA sequence or various *ESR1* mutations. Cells were grown in steroid hormone-deprived conditions and treated with DMSO or 10 nM β-estradiol (E2) for 24 h. Relative luciferase units were measured using a Dual-Glo® Luciferase Assay (Promega) and Firefly luciferase activity was normalized to Renilla luciferase activity. Data are representative of three independent experiments; error bars indicate the standard deviation of three replicates.

Supplementary Figure 3 – Part 2 *PIK3CA* tissue vs plasma status; *ESR1* plasma status

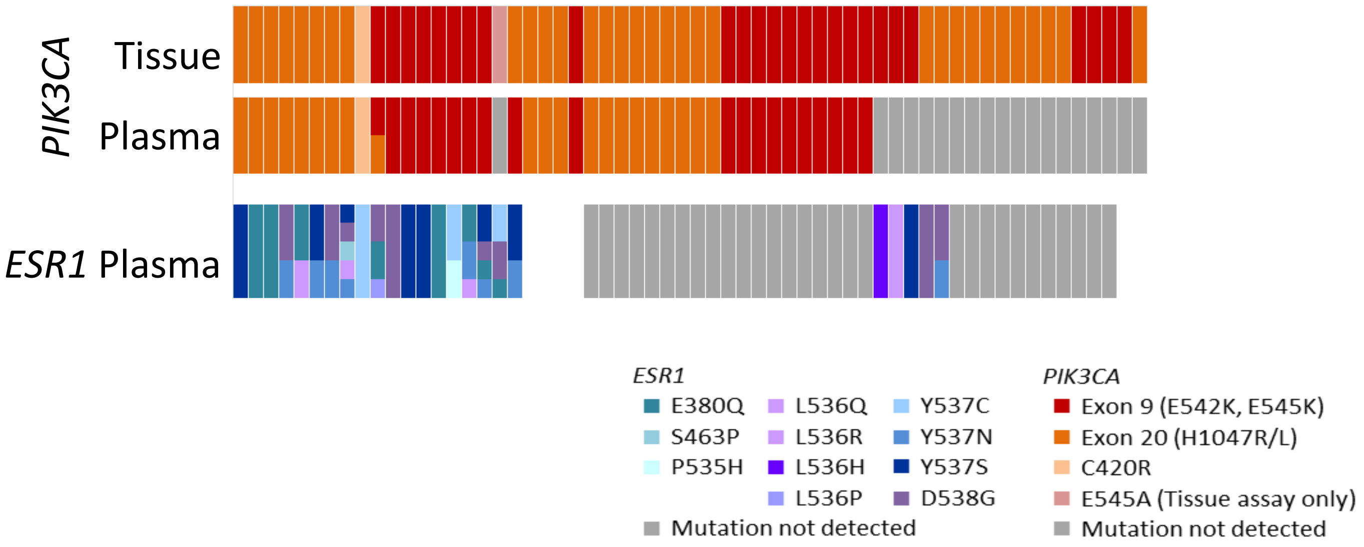

Supplementary Figure 3 *PIK3CA* and *ESR1* mutation analysis of matched tumor tissue and plasma samples from n=60 patients from part 2 of the clinical study. Mutations are colored according to exon (*PIK3CA*) or amino acid (*ESR1*). Grey indicates mutations assessed were negative, and blank indicates data not available.

Supplementary Figure 4
Kaplan-Meier plots of progression free survival by treatment

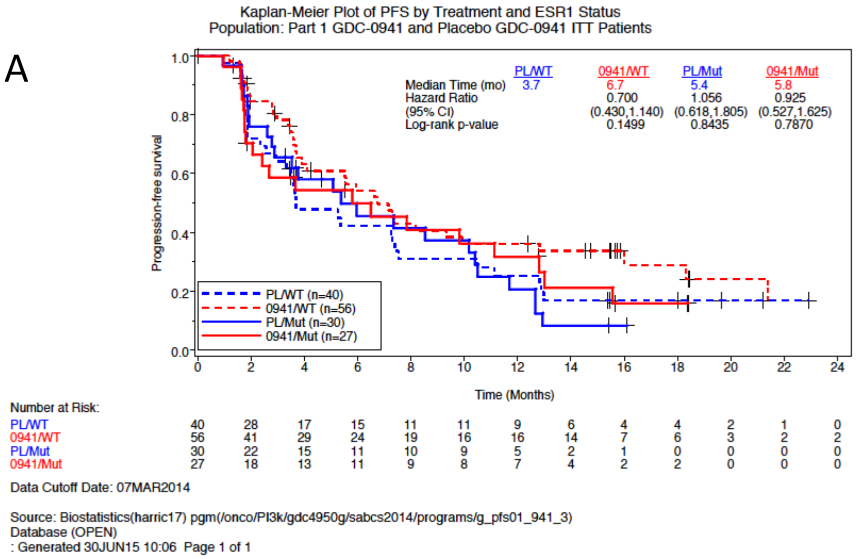

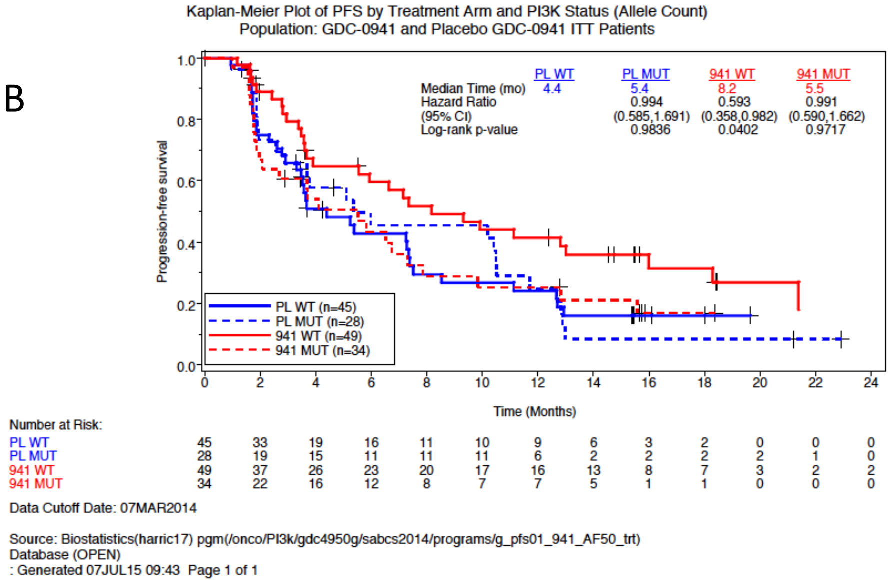

Supplementary Figure 4 Kaplan-Meier plots of progression-free survival in part 1 patients. PFS of the fulvestrant control arm (PL, blue) vs fulvestrant + pictilicib (0941, red) for patients with wildtype (WT, dashed line) or mutant (solid line) *ESR1* **(A)** and *PIK3CA* **(B).** Hazard ratios are relative to PL/WT for both plots.

Supplementary Figure 5
Intrapatient patterns of mutant allele frequency changes over time

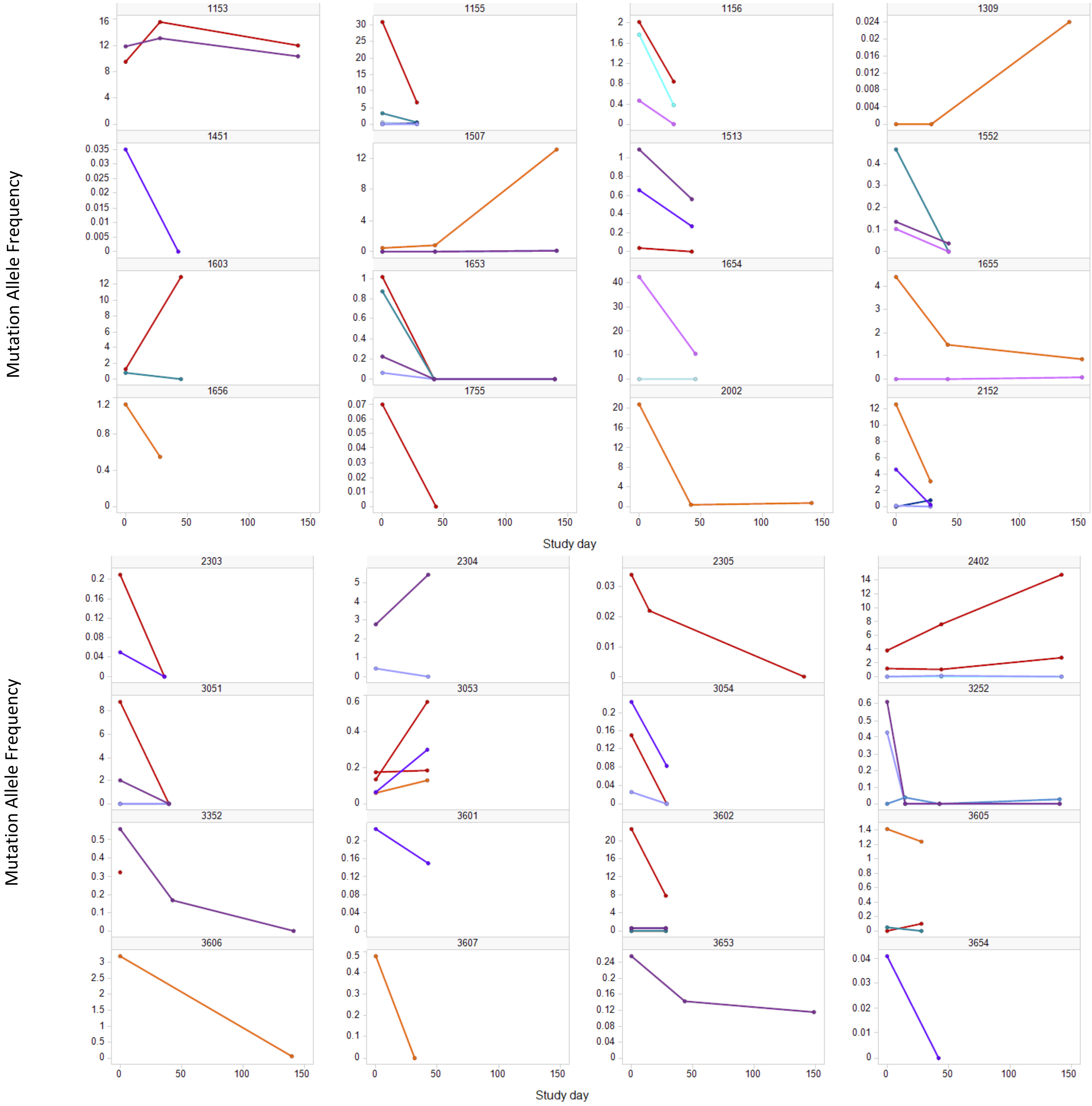

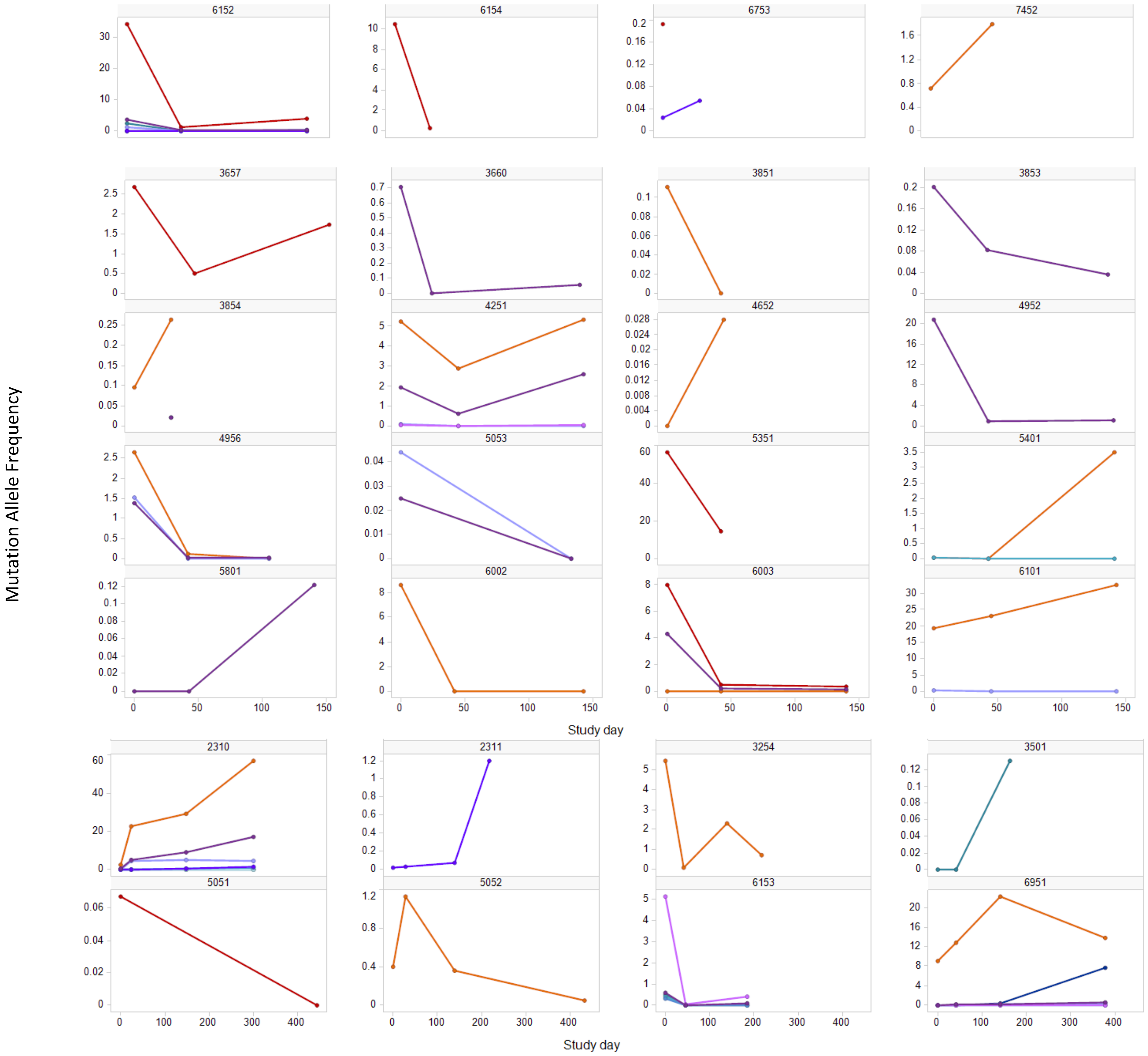
6152
6154
6753
7452
3657
3660
3851
3853
3854
4251
4652
4952
4956
5053
5351
5401
5801
6002
6003
6101
Study day
2310
2311
3254
3501
5051
5052
6153
6951
Study day
Mutation Allele Frequency

Supplemental Figure 5 Absolute *ESR1* and *PIK3CA* mutant allele frequency changes in 60 patients with at least one mutation detected in at least one time point.

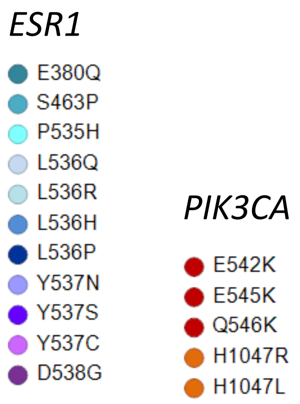

Supplementary Figure 6
Mutant allele frequency changes and tumor response

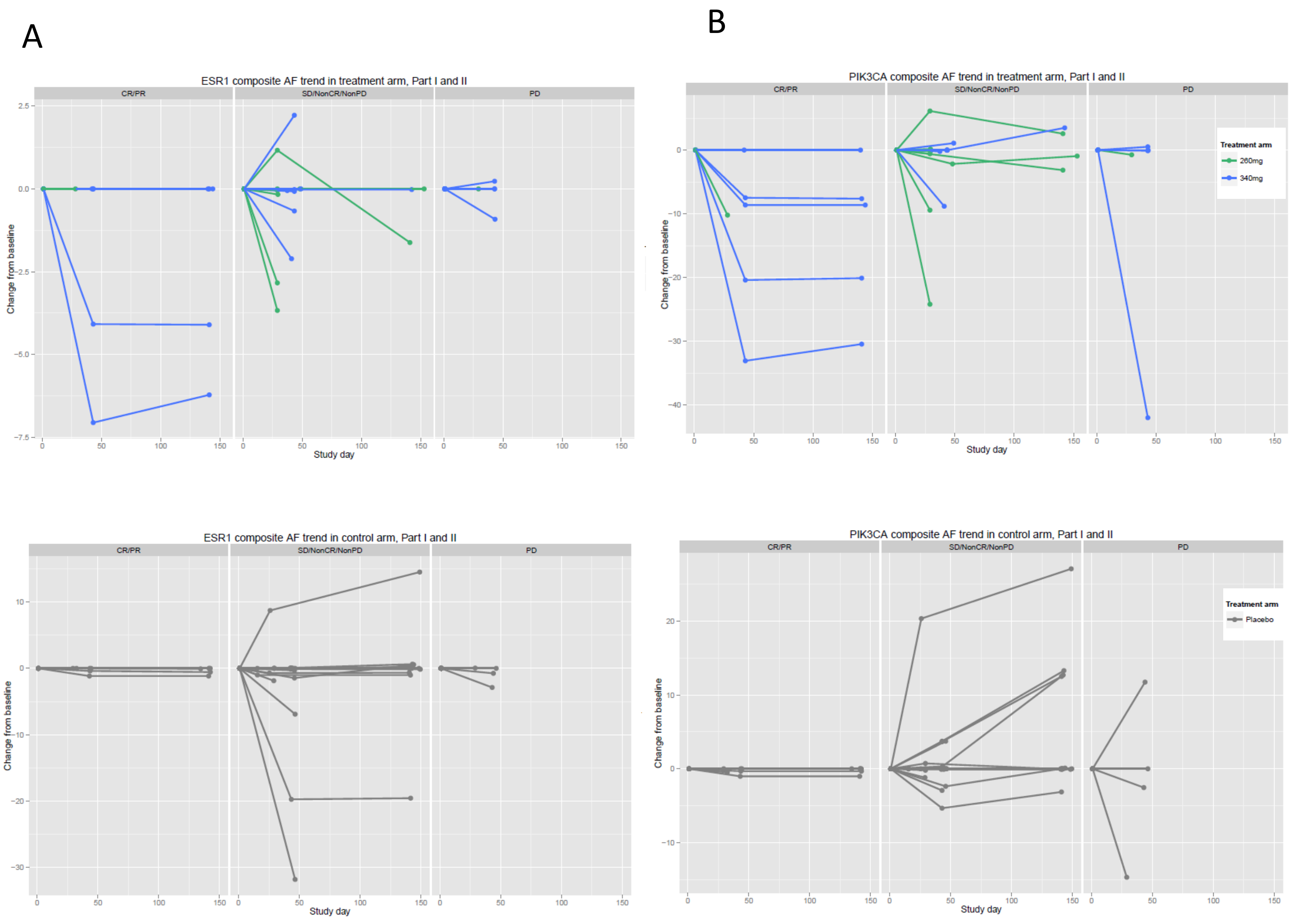

Supplementary Figure 6 **(A)** *ESR1* and (**B**) *PIK3CA* ctDNA mutation AF changes while on part 1 (green) or part 2(blue) pictilisib + fulvestrant or placebo + fulvestrant (grey) treatment arms. Patients are binned according to their best clinical response. Each line represents one patient with the absolute change of allele frequency relative to baseline (study day 0) plotted for 2 or 3 time-points. The mutation allele frequency sum is plotted for patients with multiple ESR1 or PIK3CA mutations.

Supplementary Table 1: Baseline ctDNA mutation prevalence in molecular subsets

Part 1 Randomized GDC−0941 and Placebo GDC−0941 Patients

		ESR1 Mutation Prevalence n / total (%)
PIK3CA Status, ctDNA		
	Wild-type	23 / 91 (25.3%)
	Mutation	34 / 62 (54.8%)
PIK3CA Status, tissue		
	Wild-type	23 / 76 (30.3%)
	Mutation	28 / 63 (44.4%)
Luminal Status		
	Luminal A	41 / 99 (41.4%)
	Luminal B	14 / 44 (31.8%)
	Other	2 / 10 (20%)
Progesterone Status		
	Positive	42 / 109 (38.5%)
	Negative	10 / 30 (33.3%)

Part 2 Randomized GDC−0941 and Placebo GDC−0941 Patients

		ESR1 Mutation Prevalence n / total (%)
PIK3CA Status, tissue		
	Wild-type	0 / 0
	Mutation	24 / 54 (44.4%)

Supplementary Table 2: BEAMing assay performance for *PIK3CA* from Part I samples

		PIK3CA Plasma		
		Mutant	Wild-type	
PIK3CA Tissue	Mutant	48	14	Sensitivity 77%
	Wild-type	5	71	Specificity 93%
		Concordance 86%		

N=142 matched tissue and plasma
4 pairs excluded due to different *PIK3CA* mutations detected in plasma vs tissue (2) or differences in assay coverage (2)

Supplementary Table 3: *ESR1* mutations detected in tissue

ESR1 Tissue status	*ESR1* ctDNA status
Y537N (5.3%) E380Q (0.029%) L536Q (23.7%)	**E380Q mutant (3.278%) Y537N mutant (0.088%) L536Q mutant (0.443%)**
Y537C (1.9%) E380Q (6.7%)	**Y537C mutant (5.129%)** D538G mutant (0.576%) **E380Q mutant (0.489%)** S463P mutant (0.431%) L536H mutant (0.314%)
D538G (20.1%)	**D538G mutant (1.678%)** Y537C mutant (1.336%) E380Q mutant (0.072%)
D538G (26.7%)	**D538G mutant (1.678%)** Y537N mutant (0.19%)
D538G (0.49%)	**D538G mutant (5.225%)** E380Q mutant (0.022%)
P535H (5.7%)	**P535H mutant (1.781%)** Y537C mutant (0.465%)
D538G (45%)	**D538G mutant (0.794%)**
E380Q (0.025%)	**E380Q mutant (0.024%)**
E380Q (0.59%)	**E380Q mutant (0.032%)**
E380Q (18.1%)	**E380Q mutant (0.064%)**
Y537S (17.5%)	**Y537S mutant (2.812%)**
E380Q (0.1%)	Wild-type
Y537C (0.15%)	Wild-type
Y537N (20.1%)	Wild-type
Y537C (0.17%)	Wild-type

Supplementary Table 4: *ESR1* mutation status concordance between tissue and plasma

			ESR1 Plasma		
			Mutant	Wild-type	Concordance
ESR1 Tissue	Overall N=98	Mutant	11	4	31%
		Wild-type	64	19	
	Primary N=42	Mutant	0	0	5%
		Wild-type	40	2	
	Metastatic N=47	Mutant	11	4	47%
		Wild-type	21	11	
	Tissue collected post AI N=21	Mutant	9	3	57%
		Wild-type	6	3	
	Tissue collected pre AI N=77	Mutant	2	1	23%
		Wild-type	58	16	

Supplementary Table 5: Demographic and Baseline Characteristics by *ESR1* Status; Part 1 Randomized GDC−0941 and Placebo GDC−0941 Patients

		ESR1 Wild Type<br>N=96	ESR1 Mutant<br>N=57	All Patients<br>N=153
ESR1 Mutations				
	n	96	57	153
	0	96 (100.0%)	0	96 (62.7%)
	1	0	34 (59.6%)	34 (22.2%)
	2	0	13 (22.8%)	13 (8.5%)
	3	0	7 (12.3%)	7 (4.6%)
	4	0	1 (1.8%)	1 (0.7%)
	5	0	2 (3.5%)	2 (1.3%)
Age				
	n	96	57	153
	Mean (SD)	62.0 (10.3)	59.7 (10.3)	61.1 (10.4)
	Median	62.5	60.0	62.0
	Min - Max	40 – 90	36 – 82	36 – 90
Age group (yr)				
	n	96	57	153
	< 65	58 (60.4%)	40 (70.2%)	98 (64.1%)
	>= 65	38 (39.6%)	17 (29.8%)	55 (35.9%)
Baseline Weight (kg)				
	n	95	56	151
	Mean (SD)	69.5 (14.9)	71.8 (16.2)	70.3 (15.4)
	Median	68.0	68.1	68.0
	Min - Max	38 – 119	39 – 115	38 – 119
Race				
	n	96	57	153
	Asian	7 (7.3%)	5 (8.8%	12 (7.8%)
	Black or African American	1 (1.0%)	1 (1.8%)	2 (1.3%)
	White	85 (88.5%)	49 (86.0%)	134 (87.6%)
	Other	3 (3.1%)	1 (1.8%)	4 (2.6%)
	Multiple	0	1 (1.8%)	1 (0.7%)
Region				
	n	96	57	153
	Asia	17 (17.7%)	14 (24.6%)	31 (20.3%)
	Europe	47 (49.0%)	22 (38.6%)	69 (45.1%)
	North America	27 (28.1%)	19 (33.3%)	46 (30.1%)
	Other	5 (5.2%)	2 (3.5%)	7 (4.6%)

US			
n	96	57	153
US	27 (28.1%)	19 (33.3%)	46 (30.1%)
ex	69 (71.9%)	38 (66.7%)	107 (69.9%)
Baseline ECOG Score			
n	95	57	152
0	66 (69.5%)	33 (57.9%)	99 (65.1%)
1	29 (30.5%)	24 (42.1%)	53 (34.9%)
Primary/Secondary Resistance (IVRS)			
n	96	57	153
Primary Resistance	46 (47.9%)	30 (52.6%)	76 (49.7%)
Secondary Resistance	50 (52.1%)	27 (47.4%)	77 (50.3%)
Resistance to Prior AI (BOLERO2)			
n	96	57	153
Yes	22 (22.9%)	9 (15.8%)	31 (20.3%)
No	74 (77.1%)	48 (84.2%)	122 (79.7%)
Measurable Disease (IVRS)			
n	96	57	153
Measurable	58 (60.4%)	28 (49.1%)	86 (56.2%)
Non−Measurable	38 (39.6%)	29 (50.9%)	67 (43.8%)
Measurable Disease (Derived)			
n	96	57	153
Measurable	53 (55.2%)	33 (57.9%)	86 (56.2%)
Non−Measurable	43 (44.8%)	24 (42.1%)	67 (43.8%)
ER Status			
n	96	57	153
Positive	93 (96.9%)	55 (96.5%)	148 (96.7%)
Negative	1 (1.0%)	0	1 (0.7%)
Status Unknown	2 (2.1%)	2 (3.5%)	4 (2.6%)
Her2			
n	96	57	153
Equivocal	0	1 (1.8%)	1 (0.7%)
Negative	92 (95.8%)	52 (91.2%)	144 (94.1%)
Positive	2 (2.1%)	1 (1.8%)	3 (2.0%)
Unknown	2 (2.1%)	3 (5.3%)	5 (3.3%)
PR Status			
n	96	57	153
Positive	67 (69.8%)	42 (73.7%)	109 (71.2%)
Negative	20 (20.8%)	10 (17.5%)	30 (19.6%)
Status Unknown	9 (9.4%)	5 (8.8%)	14 (9.2%)
PIK3CA Mutation Status			
n	96	57	153

Positive	35 (36.5%)	28 (49.1%)	63 (41.2%)
Wild Type	53 (55.2%)	23 (40.4%)	76 (49.7%)
Unknown	8 (8.3%)	6 (10.5%)	14 (9.2%)
Number of Previous Therapies in Metastatic Setting			
n	96	57	153
0	27 (28.1%)	13 (22.8%)	40 (26.1%)
1	38 (39.6%)	28 (49.1%)	66 (43.1%)
2	23 (24.0%)	12 (21.1%)	35 (22.9%)
3+	8 (8.3%)	4 (7.0%)	12 (7.8%)
Bone−only Disease			
n	96	57	153
Yes	24 (25.0%)	11 (19.3%)	35 (22.9%)
No	72 (75.0%)	46 (80.7%)	118 (77.1%)
Baseline Tumor Burden (Continuous)			
n	96	57	153
Mean (SD)	23.66 (26.07)	27.34 (33.73)	25.03 (29.11)
Median	16.30	20.00	17.00
Min − Max	0.0 − 87.0	0.0 − 184.1	0.0 − 184.1
Baseline Tumor Burden (Categorical)			
n	96	57	153
>= Median	48 (50.0%)	30 (52.6%)	78 (51.0%)
< Median	48 (50.0%)	27 (47.4%)	75 (49.0%)
Number of Metastatic Sites			
n	96	57	153
0	5 (5.2%)	1 (1.8%)	6 (3.9%)
1	37 (38.5%)	13 (22.8%)	50 (32.7%)
2	30 (31.3%)	24 (42.1%)	54 (35.3%)
3+	24 (25.0%)	19 (33.3%)	43 (28.1%)
Setting of Last CA Therapy			
n	96	57	153
Adjuvant	22 (22.9%)	12 (21.1%)	34 (22.2%)
Metastatic	72 (75.0%)	45 (78.9%)	117 (76.5%)
Other	2 (2.1%)	0	2 (1.3%)
Previous Chemotherapy			
n	96	57	153
No	33 (34.4%)	18 (31.6%)	51 (33.3%)
Yes − Adjuvant/Neo−Adjuvant	43 (44.8%)	28 (49.1%)	71 (46.4%)
Yes − Metastatic	20 (20.8%)	11 (19.3%)	31 (20.3%)
Number of Previous Therapies			
n	96	57	153
1	4 (4.2%)	1 (1.8%)	5 (3.3%)
2	13 (13.5%)	8 (14.0%)	21 (13.7%)

3+	79 (82.3%)	48 (84.2%)	127 (83.0%)
Prior Tamoxifen Use			
n	96	57	153
Yes	52 (54.2%)	26 (45.6%)	78 (51.0%)
No	44 (45.8%)	31 (54.4%)	75 (49.0%)
Number of Prior Tamoxifen Regimens			
n	96	57	153
0	44 (45.8%)	31 (54.4%)	75 (49.0%)
1	51 (53.1%)	26 (45.6%)	77 (50.3%)
2	1 (1.0%)	0	1 (0.7%)
Prior AI Use			
n	96	57	153
Yes	96 (100.0%)	57 (100.0%)	153 (100.0%)
Number of Prior AI Regimens			
n	96	57	153
1	71 (74.0%)	47 (82.5%)	118 (77.1%)
2	24 (25.0%)	10 (17.5%)	34 (22.2%)
3	1 (1.0%)	0	1 (0.7%)
Prior Endocrine Therapy Use			
n	96	57	153
Yes	96 (100.0%)	57 (100.0%)	153 (100.0%)
Number of Prior Endocrine Therapy Regimens			
n	96	57	153
1	35 (36.5%)	27 (47.4%)	62 (40.5%)
2	45 (46.9%)	24 (42.1%)	69 (45.1%)
3	14 (14.6%)	6 (10.5%)	20 (13.1%)
4	2 (2.1%)	0	2 (1.3%)

Data Cutoff Date: 07MAR2014
Data Extract Date: 15SEP2014

Supplementary Table 6: Demographic and Baseline Characteristics by *PIK3CA* Status; Part 1 Randomized GDC−0941 and Placebo GDC−0941 Patients

		PIK3CA Wild Type N=94	PIK3CA Mutant N=62	All Patients N=156
ESR1 Mutations				
	n	91	62	153
	0	68 (74.7%)	28 (45.2%)	96 (62.7%)
	1	13 (14.3%)	21 (33.9%)	34 (22.2%)
	2	7 (7.7%)	6 (9.7%)	13 (8.5%)
	3	1 (1.1%)	6 (9.7%)	7 (4.6%)
	4	1 (1.1%)	0	1 (0.7%)
	5	1 (1.1%)	1 (1.6%)	2 (1.3%)
Age				
	n	94	62	156
	Mean (SD)	62.0 (10.8)	59.4 (9.4)	61.0 (10.3)
	Median	63.0	60.5	62.0
	Min − Max	40 − 90	36 − 80	36 − 90
Age group (yr)				
	n	94	62	156
	< 65	57 (60.6%)	44 (71.0%)	101 (64.7%)
	>= 65	37 (39.4%)	18 (29.0%)	55 (35.3%)
Baseline Weight (kg)				
	n	92	62	154
	Mean (SD)	71.6 (15.3)	68.1 (15.1)	70.2 (15.3)
	Median	68.8	64.8	67.8
	Min − Max	47 − 119	38 − 106	38 − 119
Race				
	n	94	62	156
	Asian	6 (6.4%)	7 (11.3%)	13 (8.3%)
	Black or African American	0	2 (3.2%)	2 (1.3%)
	White	85 (90.4%)	51 (82.3%)	136 (87.2%)
	Other	2 (2.1%)	2 (3.2%)	4 (2.6%)
	Multiple	1 (1.1%)	0	1 (0.6%)
Region				
	n	94	62	156
	Asia	16 (17.0%)	18 (29.0%)	34 (21.8%)
	Europe	42 (44.7%)	27 (43.5%)	69 (44.2%)
	North America	30 (31.9%)	16 (25.8%)	46 (29.5%)
	Other	6 (6.4%)	1 (1.6%)	7 (4.5%)
US				
	n	94	62	156
	US	30 (31.9%)	16 (25.8%)	46 (29.5%)

	ex	64 (68.1%)	46 (74.2%)	110 (70.5%)
Baseline ECOG Score				
	n	93	62	155
	0	61 (65.6%)	38 (61.3%)	99 (63.9%)
	1	32 (34.4%)	24 (38.7%)	56 (36.1%)
Primary/Secondary Resistance (IVRS)				
	n	94	62	156
	Primary Resistance	43 (45.7%)	34 (54.8%)	77 (49.4%)
	Secondary Resistance	51 (54.3%)	28 (45.2%)	79 (50.6%)
Resistance to Prior AI (BOLERO2)				
	n	94	62	156
	Yes	21 (22.3%)	11 (17.7%)	32 (20.5%)
	No	73 (77.7%)	51 (82.3%)	124 (79.5%)
Measurable Disease (IVRS)				
	n	94	62	156
	Measurable	55 (58.5%)	32 (51.6%)	87 (55.8%)
	Non−Measurable	39 (41.5%)	30 (48.4%)	69 (44.2%)
Measurable Disease (Derived)				
	n	94	62	156
	Measurable	54 (57.4%)	33 (53.2%)	87 (55.8%)
	Non−Measurable	40 (42.6%)	29 (46.8%)	69 (44.2%)
ER Status				
	n	94	62	156
	Positive	93 (98.9%)	58 (93.5%)	151 (96.8%)
	Negative	0	1 (1.6%)	1 (0.6%)
	Status Unknown	1 (1.1%)	3 (4.8%)	4 (2.6%)
Her2				
	n	94	62	156
	Equivocal	1 (1.1%)	0	1 (0.6%)
	Negative	88 (93.6%)	59 (95.2%)	147 (94.2%)
	Positive	2 (2.1%)	1 (1.6%)	3 (1.9%)
	Unknown	3 (3.2%)	2 (3.2%)	5 (3.2%)
PR Status				
	n	94	62	156
	Positive	64 (68.1%)	46 (74.2%)	110 (70.5%)
	Negative	21 (22.3%)	10 (16.1%)	31 (19.9%)
	Status Unknown	9 (9.6%)	6 (9.7%)	15 (9.6%)
Number of Previous Therapies in Metastatic Setting				
	n	94	62	156
	0	23 (24.5%)	18 (29.0%)	41 (26.3%)
	1	42 (44.7%)	25 (40.3%)	67 (42.9%)
	2	21 (22.3%)	15 (24.2%)	36 (23.1%)
	3+	8 (8.5%)	4 (6.5%)	12 (7.7%)
Visceral Disease				
	n	94	62	156

Yes	51 (54.3%)	31 (50.0%)	82 (52.6%)
No	43 (45.7%)	31 (50.0%)	74 (47.4%)
Resistance to Prior AI (Derived)			
n	94	62	156
Primary	35 (37.2%)	25 (40.3%)	60 (38.5%)
Secondary	58 (61.7%)	37 (59.7%)	95 (60.9%)
Unknown	1 (1.1%)	0	1 (0.6%)
Bone−only Disease			
n	94	62	156
Yes	18 (19.1%)	18 (29.0%)	36 (23.1%)
No	76 (80.9%)	44 (71.0%)	120 (76.9%)
Baseline Tumor Burden (Continuous)			
n	94	62	156
Mean (SD)	26.40 (30.82)	22.72 (26.37)	24.93 (29.10)
Median	18.50	15.00	17.00
Min − Max	0.0 − 184.1	0.0 − 97.0	0.0 − 184.1
Baseline Tumor Burden (Categorical)			
n	94	62	156
>= Median	50 (53.2%)	29 (46.8%)	79 (50.6%)
< Median	44 (46.8%)	33 (53.2%)	77 (49.4%)
Number of Metastatic Sites			
n	94	62	156
0	5 (5.3%)	1 (1.6%)	6 (3.8%)
1	30 (31.9%)	21 (33.9%)	51 (32.7%)
2	31 (33.0%)	24 (38.7%)	55 (35.3%)
3+	28 (29.8%)	16 (25.8%)	44 (28.2%)
Setting of Last CA Therapy			
n	94	62	156
Adjuvant	19 (20.2%)	16 (25.8%)	35 (22.4%)
Metastatic	74 (78.7%)	45 (72.6%)	119 (76.3%)
Other	1 (1.1%)	1 (1.6%)	2 (1.3%)
Previous Chemotherapy			
n	94	62	156
No	35 (37.2%)	18 (29.0%)	53 (34.0%)
Yes − Adjuvant/Neo−Adjuvant	39 (41.5%)	33 (53.2%)	72 (46.2%)
Yes − Metastatic	20 (21.3%)	11 (17.7%)	31 (19.9%)
Number of Previous Therapies			
n	94	62	156
1	5 (5.3%)	0	5 (3.2%)
2	13 (13.8%)	8 (12.9%)	21 (13.5%)
3+	76 (80.9%)	54 (87.1%)	130 (83.3%)
Prior Tamoxifen Use			
n	94	62	156
Yes	48 (51.1%)	32 (51.6%)	80 (51.3%)
No	46 (48.9%)	30 (48.4%)	76 (48.7%)

Number of Prior Tamoxifen Regimens			
n	94	62	156
0	46 (48.9%)	30 (48.4%)	76 (48.7%)
1	47 (50.0%)	32 (51.6%)	79 (50.6%)
2	1 (1.1%)	0	1 (0.6%)
Prior AI Use			
n	94	62	156
Yes	94 (100.0%)	62 (100.0%)	156 (100.0%)
Number of Prior AI Regimens			
n	94	62	156
1	74 (78.7%)	46 (74.2%)	120 (76.9%)
2	19 (20.2%)	16 (25.8%)	35 (22.4%)
3	1 (1.1%)	0	1 (0.6%)
Prior Endocrine Therapy Use			
n	94	62	156
Yes	94 (100.0%)	62 (100.0%)	156 (100.0%)
Number of Prior Endocrine Therapy Regimens			
n	94	62	156
1	41 (43.6%)	21 (33.9%)	62 (39.7%)
2	38 (40.4%)	34 (54.8%)	72 (46.2%)
3	13 (13.8%)	7 (11.3%)	20 (12.8%)
4	2 (2.1%)	0	2 (1.3%)

Data Cutoff Date: 07MAR2014
Data Extract Date: 15SEP2014

Supplementary Table 7: Droplet digital PCR (ddPCR) assay sequences for tissue assessment of *ESR1* mutation status

*ESR1*ddPCR Assay	Forward Primer Sequence	Reverse Primer Sequence	WT Probe Sequence	Mutant Probe Sequence
E380Q	GGATTTGACCCTCCATGA	AGACCAATCATCAGGATCTC	ACATTCTAGAAGGTGGACC	ACATTGTAGAAGGTGGACC
P535H	ATCTGTACAGCATGAAGTG	GTCCAGCATCTCCAGC	TAGAGGGGCACCACG	TAGAGGTGCACCACGT
L536H	GTACAGCATGAAGTGCAA	GCGTCCAGCATCTCC	TCATAGAGGGGCACCA	TCATAGTGGGGCACCA
L536P	GTACAGCATGAAGTGCAA	GCGTCCAGCATCTCC	TCATAGAGGGGCACCA	TCATAGGGGGGCACC
L536Q	GTACAGCATGAAGTGCAA	GCGTCCAGCATCTCC	TGGTGCCCCTCTATGA	TGGTGCCCCAGTATGA
L536R	GTACAGCATGAAGTGCAA	GCGTCCAGCATCTCC	TGCCCCTCTATGACCTG	TGCCCCGCTATGACC
Y537C	GTACAGCATGAAGTGCAA	GGGCGTCCAGCATC	CCCCTCTATGACCTGCT	TGCCCCTCTGTGACC
Y537N	GTACAGCATGAAGTGCAA	GGCGTCCAGCATCTC	CCCCTCTATGACCTGC	AGGTCATTGAGGGGCA
Y537S	GTACAGCATGAAGTGCAA	GGGCGTCCAGCATC	AGCAGGTCATAGAGGGG	AGCAGGTCAGAGAGGG
D538G	TACAGCATGAAGTGCAAG	TGGGCGTCCAGCA	CCCCTCTATGACCTGCT	TCTATGGCCTGCTGCT

The BioRad QX200™ Droplet Digital PCR System is used for tissue DNA assessment of *ESR1* mutation status.

Supplementary Table 8: PAM50 signature gene set

Gene	Accession Number
ACTR3B	NM_001040135.1
ANLN	NM_018685.2
BAG1	NM_004323.3
BCL2	NM_000657.2
BIRC5	NM_001168.2
BLVRA	NM_000712.3
CCNB1	NM_031966.2
CCNE1	NM_001238.1
CDC20	NM_001255.2
CDC6	NM_001254.3
CDH3	NM_001793.4
CENPF	NM_016343.3
CEP55	NM_018131.3
CXXC5	NM_016463.7
EGFR	NM_201282.1
ERBB2	NM_004448.2
ESR1	NM_000125.2
EXO1	NM_003686.3
FGFR4	NM_002011.3
FOXA1	NM_004496.2
FOXC1	NM_001453.1
GPR160	NM_014373.2
GRB7	NM_005310.2
KIF2C	NM_006845.3
KRT14	NM_000526.4
KRT17	NM_000422.2
KRT5	NM_000424.2
MAPT	NM_016834.3
MDM2	NM_006878.2
MELK	NM_014791.2
MIA	NM_006533.1
MKI67	NM_002417.2
MLPH	NM_024101.4
MMP11	NM_005940.3
MYBL2	NM_002466.2
MYC	NM_002467.3
NAT1	NM_001160174.1
NDC80	NM_006101.2
NUF2	NM_031423.3
ORC6	NM_014321.2
PGR	NM_000926.2
PHGDH	NM_006623.2
PTTG1	NM_004219.2
RRM2	NM_001034.1
SFRP1	NM_003012.3
SLC39A6	NM_012319.2
TMEM45B	NM_138788.3
TYMS	NM_001071.1
UBE2C	NM_181803.1
UBE2T	NM_014176.3
